# Coordinated control of genome–nuclear lamina interactions by topoisomerase 2B and lamin B receptor

**DOI:** 10.1093/nar/gkaf964

**Published:** 2025-09-26

**Authors:** Tom van Schaik, Mikhail Magnitov, Marcel de Haas, Jeremie Breda, Elzo de Wit, Anna G Manjon, René H Medema, Henrike Johanna Gothe, Vassilis Roukos, Adam J Buckle, Catherine Naughton, Nick Gilbert, Bas van Steensel, Stefano G Manzo

**Affiliations:** Division of Gene Regulation, Netherlands Cancer Institute, 1066 CX Amsterdam, The Netherlands; Oncode Institute, The Netherlands; Division of Gene Regulation, Netherlands Cancer Institute, 1066 CX Amsterdam, The Netherlands; Division of Gene Regulation, Netherlands Cancer Institute, 1066 CX Amsterdam, The Netherlands; Oncode Institute, The Netherlands; Division of Molecular Genetics, Netherlands Cancer Institute, 1066 CX Amsterdam, The Netherlands; Division of Gene Regulation, Netherlands Cancer Institute, 1066 CX Amsterdam, The Netherlands; Oncode Institute, The Netherlands; Division of Gene Regulation, Netherlands Cancer Institute, 1066 CX Amsterdam, The Netherlands; Division of Cell Biology, Netherlands Cancer Institute, 1066 CX Amsterdam, The Netherlands; Division of Cell Biology, Netherlands Cancer Institute, 1066 CX Amsterdam, The Netherlands; Princess Maxima Center, 3584 CS Utrecht, The Netherlands; Institute of Molecular Biology gGmbH, 55128 Mainz, Germany; Institute of Molecular Biology gGmbH, 55128 Mainz, Germany; Department of Biology, Medical School, University of Patras, 26500 Patras, Greece; Institute of Genetics and Cancer, MRC Human Genetics Unit, The University of Edinburgh, EH4 1QY Edinburgh; Institute of Genetics and Cancer, MRC Human Genetics Unit, The University of Edinburgh, EH4 1QY Edinburgh; Institute of Genetics and Cancer, MRC Human Genetics Unit, The University of Edinburgh, EH4 1QY Edinburgh; Division of Gene Regulation, Netherlands Cancer Institute, 1066 CX Amsterdam, The Netherlands; Oncode Institute, The Netherlands; Division of Molecular Genetics, Netherlands Cancer Institute, 1066 CX Amsterdam, The Netherlands; Division of Gene Regulation, Netherlands Cancer Institute, 1066 CX Amsterdam, The Netherlands; Oncode Institute, The Netherlands; Department of Biosciences, University “La Statale” of Milan, 20133 Milan, Italy

## Abstract

Lamina-associated domains (LADs) are megabase-sized genomic regions anchored to the nuclear lamina (NL). Factors controlling the interactions of the genome with the NL have largely remained elusive. Here, we identified DNA topoisomerase 2 beta (TOP2B) as a regulator of these interactions. TOP2B binds predominantly to inter-LAD (iLAD) chromatin and its depletion results in a partial loss of genomic partitioning between LADs and iLADs, suggesting that this enzyme might protect specific iLADs from interacting with the NL. TOP2B depletion affects LAD interactions with lamin B receptor (LBR) more than with lamins. LBR depletion phenocopies the effects of TOP2B depletion, despite the different positioning of the two proteins in the genome. This suggests a complementary mechanism for organizing the genome at the NL. Indeed, co-depletion of TOP2B and LBR causes partial LAD/iLAD inversion, reflecting changes typical of oncogene-induced senescence. We propose that a coordinated axis controlled by TOP2B in iLADs and LBR in LADs maintains the partitioning of the genome between the NL and the nuclear interior.

## Graphical Abstract

**Graphical Abstract:**
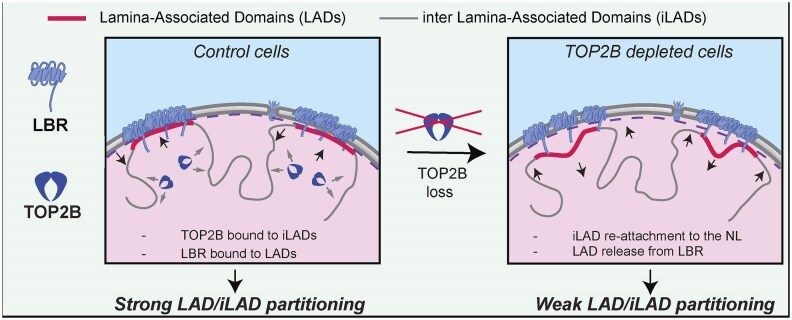

## Introduction

The typical nuclear organization of metazoan cells requires an extensive portion of the genome (up to 40%) to interact with the nuclear lamina (NL) [1–3]. Such interactions occur within extended regions of heterochromatin defined as lamina-associated domains (LADs). LADs have low gene density, harbour lowly expressed genes and heterochromatic marks such as H3K9 and H3K27 methylation, and exhibit transcriptional repressive capacity [1, 4–7]. However, despite a good characterization of LAD chromatin features, the key regulators of chromatin association with the NL remain poorly described.

The heterochromatic state of the LAD and its direct regulators have been proposed to play a role in genome–NL association. For example, H3K9me2 is a mark that is enriched at the nuclear peri-phery and is conserved across species [8]. Tethering G9a, the main H3K9me2 writer, to a specific target sequence causes its association with the NL [9]. Depletion of several factors that control heterochromatin maintenance induces LAD release from the NL [1, 2, 10, 11]. However, the casual relationship between heterochromatin homeostasis and its association with the NL has not been understood.

Heterochromatin is thought to be important for genome–NL association because it provides a scaffold of interactions for nuclear envelope (NE) tethers, transmembrane proteins that can physically tether H3K9 methylated regions to the nuclear periphery. A clear example is CEC-4 in *Caenorhabditis elegans* [12] and lamin B receptor (LBR) in mammals [13, 14], which are considered important tethers of heterochromatin. LBR has been shown to favour conventional genome organization in metazoan nuclei in synergy with the NL component lamin A [15].

The transcriptional machinery is an important negative regulator of genome–NL association. Activation of a silent gene associated with the NL promotes physical separation from this component [16–18]. Similarly, transcriptional inactivation can promote reattachment [18, 19]. Conversely, physical detachment of a repressed gene has been shown to allow its activation [20]. Furthermore, LAD remodeling during differentiation is coordinated with changes in the transcriptional program [21–24]. With a few exceptions [23], most data suggest a mutually exclusive relationship between gene activation and genome–NL association.

Transcription could shape genome–NL interactions by sequestering active chromatin away from the NL, creating an environment incompatible with NL association. Consistent with this model, in *C. elegans* the chromatin binding factor MRG1 binds active acetylated regions and sequesters them away from the NL [25]. These data suggest a competition model in which different parts of the genome compete for interaction with the NL. This model is also supported by the comparison of genome–NL interactions between haploid and diploid HAP1 cells, which showed a reduction in interaction with the NL for the diploid compared to the haploid haplotype [26]. This result supports the idea that there is a limited capacity for chromatin interactions with the NL.

One property of DNA that is emerging as a new level of regulation of chromatin structure and organization is DNA supercoiling [27, 28]. This is the ability of DNA or chromatin to change conformation following the application of torsion, stretching, or bending of the double helix. These physical forces can arise from the activity of powerful revolving machines such as polymerases, the catalytic activity of chromatin remodelers, or the binding and bending activity of DNA-binding proteins [29, 30]. Modelling and experimental data suggest that transcription-induced supercoiling may contribute to genome folding at multiple levels [28, 31, 32].

Cells have specialized enzymes to deal with DNA supercoiling, called DNA topoisomerases. These enzymes can catalyse the relaxation of chromatin by introducing transient single (type I topoisomerases) or double (type II topoisomerases) strand breaks on DNA. TOP1, TOP2A, and topoisomerase 2 beta (TOP2B) are among the most studied topoisomerases and are currently targeted in chemotherapy. While these enzymes have mainly been studied in the context of replication, transcription, and cell division [33, 34], it has recently been proposed that they may also play a role in genome organization, particularly in the context of loop extrusion machineries [35]. TOP2B positioning and cutting activity has been described at the base of CTCF-bound chromatin loops in the vicinity of active genes, suggesting that it could regulate chromatin topology between the transcription machinery and the loop-extruding cohesin complex [36–38]. Although this specific TOP2B function is likely related to DNA supercoiling modulation, TOP2B and TOP2A can also impact genome folding by regulating DNA decatenation. In yeast, in interphase, Top2 activity is counteracted by condensin loop extrusion activity and this balance regulates the level of genome entanglement [39]. TOP2 activity is also important in regulating mitotic chromosome architecture by regulating sister chromatid resolution and chromatin condensation [40, 41]. Recently, TOP2 has also been found to act during mitotic exit and its activity is required to re-establish an untangled and compartmentalized genome [42].

Despite the growing evidence that topoisomerases may be involved in 3D genome organization and folding, the relationship between these enzymes and the anchoring of the genome to the NL has not been investigated. Indeed, the literature on this topic is limited and somewhat sparse. *In vitro*nuclear reassembly in Xenopus extracts showed that TOP2 inhibitors can block the assembly of a nucleus around protein-free DNA [43, 44]. Cell-free preparations of Drosophila embryos show that lamins and DNA topoisomerases re-associate on newly assembled nuclei [45]. Top2 was also found bound to AT-rich regions that are associated to nuclear or metaphase scaffolds, highlighting a possible structural role of Top2 [46, 47]. All these data suggest a role for TOP2 in the establishment and maintenance of nuclear architecture. Interestingly, removal of the NE reduces segregation defects in topoisomerase mutants [48]. TOP2B was found to be important for the transcription of neuronal genes proximal to AT-rich regions that may overlap with LADs [49]. TOP1 was shown to be able to reduce R-loop formation for long, highly active genes proximal to the NL [50]. These data mainly point to a role of the NL as a boundary or constraint.

Apart from this disjointed knowledge on topoisomerases and the NL, a study that addresses whether and how topoisomerases control genome–NL interactions is still missing. In this work, we fill this knowledge gap. We have investigated the role of topoisomerases in genome–NL interactions and linked this to NE tethering. We found that TOP2B is a regulator of genome association with the NL that acts in coordination with the NE tether LBR to maintain proper partitioning of the genome between the NL and the nuclear interior.

## Materials and methods

### Cell lines and cell culturing

Two different hTERT immortalized RPE1 cell lines were used in this study. An RPE1 cell line that expresses the SunTag system system [18, 51] was used to perform TOP1, TOP2A, and TOP2B depletions and the mapping of heterochromatin components. This cell line was maintained in Dulbecco’s modified Eagle’s medium (DMEM; Gibco), Fetal Bovine Serum (FBS) 10%, and penicillin–streptomycin 1%. A second RPE1 cell line expressing an inducible Cas9 [52] was used for the generation of LBR knockout and all the other experiments. This cell line was maintained in DMEM:F12 (Gibco), FBS 10% and penicillin–streptomycin 1%. HCT116 were maintained in McCoy medium (Gibco) FBS 10% and penicillin–streptomycin 1%. K562 cell lines were maintained in IMDM medium (Gibco), FBS 10% and penicillin–streptomycin 1%. HCT116 TOP2B knockout cell lines were a kind gift from Vassilis Roukos. GM12878 were maintained in RPMI1640 medium (Gibco) FBS 15% and penicillin–streptomycin 1% and were a kind gift from Magda Bienko. All cell lines were routinely checked for Mycoplasma contamination.

### Antibodies used

We used the following antibodies: LMNB2 (Abcam, ab8983), LMNB1 (Abcam, ab16048), LBR (Abcam, ab32535), H3K9me3 (Diagenode, C15410193), H3K9me2 (Active Motif, 39239), H3K27me3 (Diagenode, C15410195), TOP2B (Novus Biological, NB100-40842), TOP1 (Abcam, ab109374), TOP2A (Abcam, ab52934).

### Topoisomerases depletion by RNA interference

A total of 5.4 × 10^5^ cells were transfected in suspension in a 10 cm-dish with 10 nM of short interfering RNA (siRNA) and 1:1000 of Lipofectamin RNAimax reagent (Thermo Fisher, 13778075). Forty eight hours after transfection cells were detached and expanded in four 10-cm dishes and transfected again in the same conditions. All experiments were performed at 72 h following the second round of transfection. Knockdown efficiency was routinely checked by western blot analysis. Two negative control siRNA were used: ON-TARGET plus Non-targeting Control Pool (Horizon Discovery, D-001810-10-05) and Silencer™ Select Negative Control No. 2 siRNA (Thermo Fisher, 4390846). Cell lines transfected with these two siRNAs showed an high degree of correlation for chromatin–NL contacts (*R* > 0.9), although some differences in LAD strength could be detected. When possible, and as indicated in figure legends, the two datasets were averaged together. For TOP2B depletion three TOP2B-specific siRNAs were used: Dharmacon on target plus smart pool Human TOP2B siRNA (Horizon Discovery, L-004240-00-0005), Silencer Select TOP2B siRNA s106, (Thermo Fisher, 4390824), and Silencer Select TOP2B siRNA s108 (Thermo Fisher, 4390824). When possible, and as indicated in figure legends, the datasets from the siRNA pool and s108 were averaged together. For TOP1 depletion we used Silencer Select TOP1 siRNA (Thermo Fisher, S14304) and identical protocol to TOP2B depletion. For TOP2A depletion, we used Dharmacon on target plus smart pool Human TOP2A siRNA (Horizon Discovery, L-004239-00-0005). For this specific topoisomerase we performed a single knockdown as this was sufficient to completely block cell growth.

### Generation of TOP2B knock-out in K562

For the generation of K562 TOP2BKO cells, K562 cells (ATCC, Cat #CCL-243) were first infected with lentiviral particles generated by transfection in HEK293T cells with plasmids expressing an inducible SpCas9 (pCW-Cas9; Addgene #50661) and selected in presence of 1 μg/ml puromycin to generate the stable K562_TRE_Cas9 cells. Then, K562_TRE_Cas9 cells were transduced, in presence of doxycycline to induce Cas9 expression, with viral particles generated by transfection of HEK293T cells with the plasmid pMuLE Lenti Dest Neo (Addgene #62178) expressing two single guide RNAs (sgRNAs) targeting both TOP2B exon 1 (5′-CGCGCCGCAGCCACCCGACT-3′) and exon 2 (5′-CTTCGTCCTGATACATATAT-3′). For the generation of lentiviral particles, HEK293T cells were co-transfected with the second-generation packaging vectors (psPax2, pMD2.G) and the lentiviral vector of interest. The lentiviral containing supernatant was filtered and concentrated 10–100X (Lenti-X Concentrator, Clontech). Cells were transduced with concentrated virus and 5 μg/ml polybrene (TR-1003-G, Merck) at 800 × *g* for 30 min. Transduced cells were selected with 700 μg/ml G418 for 12 days and single clones were obtained and screened for the knockout of TOP2B by immunofluorescence and western blotting.

### Topoisomerases inhibition

Merbarone (Merck-Sigma, M2070) and ICRF-193 (Merck-Sigma, I4659) were dissolved in dimethyl sulfoxide (DMSO), aliquoted and stored at −20°. Cells were treated with 200 μM of Merbarone or 10–20 μM of ICRF193 for the indicated times. As control we used drug-equivalent volumes of DMSO (0.2% final concentration).

### pA-DamID

pA-DamID maps were generated as previously described [53]. Briefly, 1 million of RPE1 cells were collected by centrifugation (500 × *g*, 3 min) and washed sequentially in ice-cold phosphate buffered saline (PBS) and digitonin wash buffer (D-Wash) (20 mM HEPES-KOH, pH 7.5, 150 mM NaCl, 0.5 mM spermidine, 0.02% digitonin, Complete Protease Inhibitor Cocktail). Cells were rotated for 2 h at 4°C in 200 μl D-Wash with 1:200 antibody dilutuon, followed by a wash step with D-Wash. When the antibody used was not produced in rabbit, cells were incubated with a solution of D-Wash buffer and Rabbit-anti-mouse IgG (1:200, Abcam, ab6709), followed by a wash step. This incubation was repeated with a 1:200 pA-Dam solution (equivalent to nearly 60 Dam units, determined by calibration against Dam enzyme from NEB, #M0222L), followed by two wash steps. Dam activity was induced by incubation for 30 min at 37°C in 100 μl D-Wash supplemented with the methyl donor SAM (80 μM) while gently shaking (500 rpm). For every condition, another 1 million cells were processed in only D-Wash and, during Dam activation, incubated with 4 units of Dam enzyme (NEB, M0222L). We use Dam-only control samples to normalize for DNA accessibility and amplification biases as described [54]. Genomic DNA was isolated and processed similarly to DamID, except that the DpnII digestion was omitted. Sixty-five base-pair reads were sequenced on HiSeq 2500 or 100 bp reads were sequenced on Novaseq platform. For Hiseq libraries library preparation was performed as previously described [53]. For Novaseq libraries we performed the following modified protocol: genomic DNA was isolated (Bioline, BIO-52067) and ∼500 ng were digested with DpnI (10 U, NEB, R0176L) in CutSmart Buffer 1× (8 h 37°C, 20 min 80°C) in a total volume of 10 μl. A-tailing was performed by adding 5 μl of the A-tailing mix (0.5 μl of Cutsmart buffer 10×, 0.25 μl Klenow 50 U/μl (NEB, M0212M), 0.05 μl dATP 100 mM, 4.2 μl H_2_O), and incubating for 30 min at 37°C followed by 20 min at 75°C. Adapters were ligated by adding 15 μl of the ligation mix [3 μl T4 Ligase Buffer 10×, 0.5 μl T4 ligase (5 U/μl, Roche, 10799009001), 0.25 μl of x-Gene Stubby Adapter 50 mM (IDT), 11.25 μl H_2_O], and incubating samples 16 h at 16°C and 10 min at 65°C. Ligation mix was purified with 1.6 volumes of custom made solution of SeedBead Magnetic Carboxylate beads (Cytiva, 65152105050350) and PEG8000 18%, Tris–HCl 10 mM, pH 8.0, ethylenediaminetetraacetic acid (EDTA) 1 mM, NaCl 1 M, Tween 0.05%. Finally, the Methyl Indexed polymerase chain reaction (PCR) was performed by mixing 4 μl of purified ligated DNA with x-Gen Dual combinatorial Indexes (IDT; 125 nM final concentration) and MyTaq RedMix (Bioline, BIO-25048) in a final volume of 40 μl. The following PCR program was used: 1× (1 min 94°C), 14× (30 s 94°C, 30 s 58°C, 30 s 72°C), 1× (2 min 72°C).

To perform calibrated pA-DamID we spiked-in RPE1 cells with 20% mouse embryonic fibrobalsts before proceeding to pA-DamID. For this specific experiment we performed LMNB1 pA-DamID as this antibody performed well in mouse cells, differently from LMNB2 antibody (see Supplementary Fig. S2C).

### Bioinformatic analysis of pA-DamID data

pA-DamID data was processed as described [55]. Briefly, the adapter sequence was trimmed with cutadapt 1.11 before mapping the remaining genomic DNA sequence to hg38 with bwa mem 0.7.17. The following steps were performed with custom R scripts. Reads with a mapping quality of at least 10 and overlapping the ends of a GATC fragment were counted in 20-kb genomic bins. Counts were normalized to 1 million reads per sample and a log_2_-ratio over the Dam control sample was calculated with a pseudo count of 1. At least two biological replicates were generated for every experimental condition and the average score was used for downstream analyses.

#### Differential LAD calling

Differential LADs calling was performed as previously described [53]. Briefly, to call differential LADs we converted the log_2_ (LMNB2: Dam) data to z‐scores (mean of zero and a standard deviation of one) to account for differences in dynamic range in pA‐DamID signals between experiments, thus keeping the data distribution identical. LADs were determined in the control sample using a hidden Markov model on the average NL interaction profile between biological replicates (https://github.com/gui11aume/HMMt). The LAD score was defined as the mean signal of the z-scaled data tracks. Significant changes were called using a modified limma‐voom approach [56]. Significance was defined as a Benjamini–Hochberg adjusted *P*‐value lower than 0.05.

#### Analysis at LAD borders

LADs as described in the section “differential LAD calling” were used to visualize the consensus NL interaction profile around LAD borders. To calculate average signal at the LAD border we used 20 kb binned genomic tracks from bTMP-seq (IP/Input) and pA-DamID [log_2_(ratio)]. The flanks of the LADs were extracted from the LAD models and used as LAD borders. LAD flanks within 50 kb of the chromosome ends were excluded as these are not actual LAD borders but simply chromosome ends. Next, for every 20 kb genomic bin, the closest LAD border was determined. Relative positioning from the closest LAD border was used to calculate the mean score and the 95% confidence interval of the mean. Taking the closest LAD border for every genomic bin ensures that each position is only considered once, even when a position is close to multiple LAD borders. For this analysis, LADs and inter-LADs (iLADs) <50 kb were excluded as these are only composed of two normalized bins (of 20 kb each). These small domains would therefore only provide a single data point at one side of the LAD border, and likely only increase the noisiness of the resulting consensus profile.

#### Calibration of pA-DamID data

Calibrated pA-DamID data was processed similar to uncalibrated data, except that the reads were aligned to the human (hg38) and mouse (mm10) genomes at the same time and that genomic bins of 250 kb were used instead of 20 kb. The latter was necessary because the number of mouse reads was limited and larger bins resulted in more stable scaling estimates. The filtering step based on a mapping quality of 10 ensured that only unique alignments to either the human or mouse genome were used in the downstream analyses. After determining the log_2_(ratio) over the Dam control using the combined genome reference, scaling factors were calculated to convert the mouse log_2_(ratios) to a z-score (see the section “different LAD scaling”). All mouse spike-ins were from the same biological sample, and this scaling ensured that all mouse profiles were normalized to the same dynamic range. Next, for each sample, the same scaling factors were used to scale the human log_2_(ratios). In contrast to uncalibrated data, with this approach quantitative differences in dynamic range between experiments due to increased or decreased protein–DNA interactions could be detected.

### *In vivo* and *ex vivo* chromatin relaxation

Bleomycin (Fisher Scientiifc, B2434-20MG) was dissolved in water, aliquoted, and stored at −20°. Treatments were performed at 10 μM and 100 nM concentrations for 3 h before processing cells for pA-DamID. For *ex vivo* experiments, cells were permeabilized according to pA-DamID protocol and then resuspended in Human topo II assay buffer (Inspiralis, HTA202) and complemented or not with 20U of Human Topo II alpha (Inspiralis, HT205) or 20U of Human Topo II beta (Inspiralis, HTB205). Enzymes activities were checked with a plasmid relaxation assay.

### bTMP-seq

bTMP-seq was performed as previously described [57] with some modifications necessary for next generation sequencing. Cells or control genomic DNA were treated with 500 μg/ml bTMP for 20 min at room temperature in the dark. bTMP was ultraviolet (UV) cross-linked to DNA at 365 nm UV 800 mJ/cm^2^ (UVP’s CL-1000). DNA was purified from cells using sodium dodecyl sulphate (SDS) and proteinase K digestion followed by phenol-chloroform-isoamyl alcohol extraction. DNA was fragmented by sonication to a range of 200–500 bp fragments. The bTMP–DNA complex in Tris 10mM-EDTA 1mM (TE) was immunoprecipitated using avidin conjugated to magnetic beads (Dynabeads MyOne Streptavidin Invitrogen, 65001) overnight at 4°C. Beads were washed sequentially for 5 min each at room temperature with TSE I (20 mM Tris, pH 8.1, 2 mM EDTA, 150 mM NaCl, 1% Triton X-100, and 0.1% SDS), TSE II (20 mM Tris, pH 8.1, 2 mM EDTA, 500 mM NaCl, 1% Triton X-100, and 0.1% SDS) and buffer III (10 mM Tris, pH 8.1, 0.25 M LiCl, 1 mM EDTA, 1% NP40 and 1% deoxycholate). Beads were then washed twice with TE buffer for 5 min. bTMP-enriched DNA was then “on-bead” end-repaired and A-tailed prior to ligation of Illumina sequencing adapters. Adapter-ligated bTMP-bound DNA was then eluted from the Dynabeads in 25 μl water by heating for 10 min at 98°C. Library preparation was continued using the NEB NEBNext^®^ UltraTM II DNA Library Prep Kit for Illumina (NEB #E7645S) starting at “Step 4: PCR amplification of adapter-ligated DNA” where 10 μl of the DNA was used. Final libraries were sized and quality controlled on a D1000 Tapestation tape (Agilent). Single-end DNA-sequencing of 50 bp read length was performed on Illumina Hi-Seq 4000 (UMC, Amsterdam).

### RNA-seq

RNA was extracted using an RNAeasy mini kit from Qiagen (#74104). One million cells were harvested, washed once in cold PBS, resuspended in 600 μl of RLT lysis buffer, and stored at −80°C. Libraries were prepared using the TruSeq^®^ RNA LT kit and TruSeq RNA Single Indexes (Illumina). We sequenced libraries with single-end 65-bp reads on a HiSeq 2500 platform. We sequenced approximately 30 million reads for every condition. Two independent biological replicates were generated. RNA-seq reads were subjected to quality control using FastQC v0.11.6. Reads were aligned to the human reference genome (GRCh38, GRCh38_no_alt_analysis_set_GCA_000001405.15; https://www.encodeproject.org/files/GRCh38_no_alt_analysis_set_GCA_000001405.15/) using STAR 2.5.4a [58] with parameters –clip5pNbases 0 –outWigStrand Unstranded. Gene-level count tables were generated while mapping using Gencode v24 primary assembly annotations.

### Analysis of publicly available data

#### CC-seq

Raw CC-seq data for RPE1 cells were downloaded from GSE136943 [59]. The data was realigned to the GRCh38 human reference genome using the terminalMapper v4.1 pipeline (https://github.com/Neale-Lab/terminalMapper) as described in the original study. The resulting base-pair resolution profiles for Watson and Crick strands were merged together, binned into 20 kb bins and normalized using the total number of reads. We used these 20 kb binned profiles to compute the difference between VP16 treated and control RPE1 cells and obtained an estimate of catalytically engaged TOP2.

#### ATMP-seq

Raw ATMP-seq data for HCT116 and GM12878 cells were downloaded from PRJNA1003820 [60]. The data was realigned to the GRCh38 human reference genome using bwa mem v0.7.17-r1188 [61]. Uniquely mapped proper read pairs (-f 2) with MAPQ > 10 were selected using [62]. Duplicate reads were filtered out using the Picard v2.25.6 “MarkDuplicates” function. Coverage tracks normalized by genomic DNA were generated using the “bamCompare” function from the deepTools v3.4.2 [63] with the “–operation” parameter set to log2 and the reads outside of the blacklist regions removed with “-bl” parameter [64].

#### Nucleosome mapping

Processed MNase and chemical nucleosome positioning data for mouse embryonic stem cells were downloaded from GSE82127 [65]. Constitutive LAD and iLAD (cLADs and ciLAD, respectively) regions in the mouse cell types were obtained from GSE17051 [66]. Nucleosome signals for MNase and chemical mapping were calculated for each cLAD and ciLAD region using the “intersect” and “groupby” functions from BEDTools v2.27.1 [67]. Statistical significance of the difference between nucleosome signals in cLAD and ciLAD regions was assessed using a *t*-test. Outlier values from top 0.5 and bottom 0.5 percentiles were excluded from plotting. Linker DNA lengths in cLAD and ciLAD regions for chemical nucleosome mapping were calculated as described in the original study [68]. First, the nucleosome positions located in the cLAD and ciLAD regions were identified using the “intersect” function from BEDTools. Second, the distance between two consecutive nucleosome centres was calculated. Lastly, in case the distance was larger than 100 bp, 147 bp was subtracted. Linker DNA lengths between 1 and 100 bp were used for plotting.

MNase-seq data for K562 and GM12878 cells was downloaded from ENCODE (GSM920557, GSM920558) [67]. For additional cell lines, MNase-seq data for Hela (GSE100401 [69]), HepG2 (GSE76344 [70]), Raji (GSE52914 [71]), T47D (GSE74308 [72]) cells were used. For GM12878 and RPE1 cell line we used Strand-seq data [73, 74]. Pile-up of the MNAse-seq or Strand-seq on cLADs (identified in [75]) was calculated using the “computeMatrix” function from deepTools v3.4.2 [63] in scale-regions mode with parameters ‘-bs’ set to 10 000, ‘–beforeRegionStartLength’ set to 500 000, ‘–regionBodyLength’ set to 1000 000, ‘–afterRegionStartLength’ set to 500 000, ‘–missingDataAsZero’, and ‘–skipZeros’. Pile-ups were visualized using the “plotHeatmap” function from deepTools.

#### Oncogene-induced senescence data

RNAseq counts for oncogene-induced senescence (OIS) experiments was downloaded from GSE130306 [76]. Genome–NL interactions maps during OIS were downloaded from GSE76605 [75] and re-aligned to GRCh38. List of SASP genes was taken from https://reactome.org/PathwayBrowser/#/R-HSA-2559582. SASP genes were compared to a subset of non SASP genes that matched the first group for expression levels. Matching was performed as in [77].

### Statistical analyses

The statistical analyses when done are indicated in the text or figure legends.

## Results

### TOP2B regulates LAD/iLAD partitioning *in vivo*

#### TOP2B controls the partitioning of the genome between LADs and iLADs

**Figure 1.**
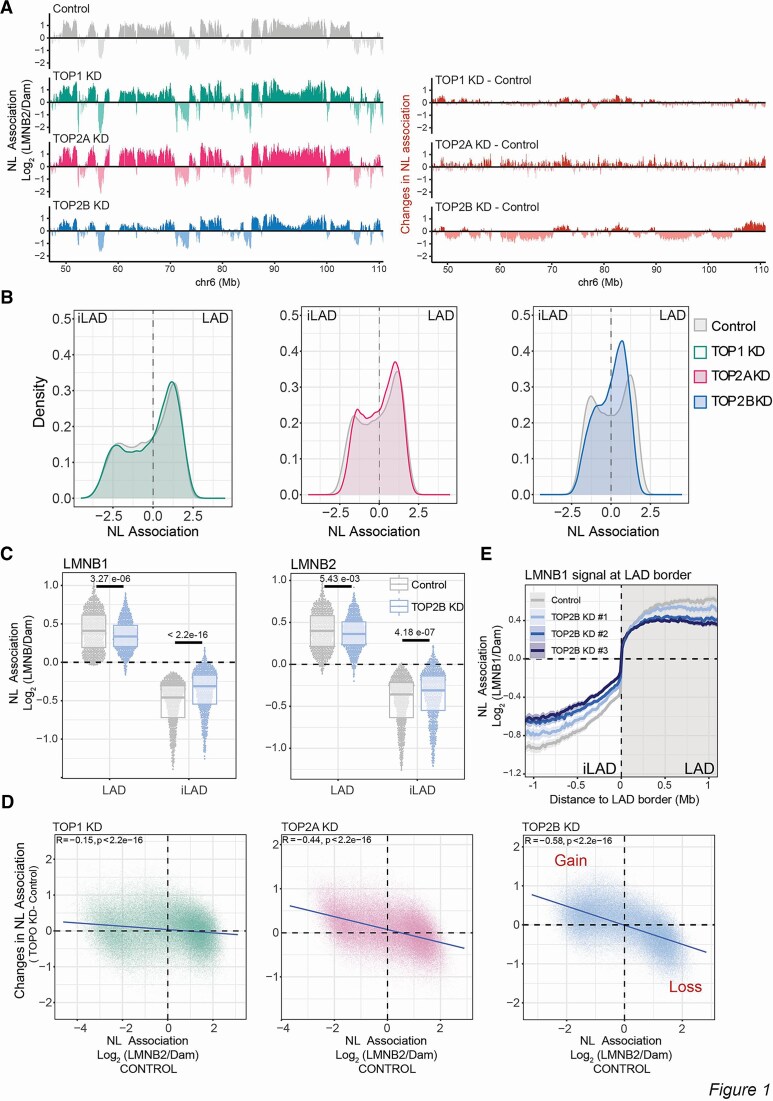
TOP2B controls genome partitioning in LAD and iLAD. (**A**) *Left:* Example genomic tracks of LMNB2 pA-DamID for control sample , TOP1, TOP2A, and TOP2B knockdowns; 20-kb bins were used. The antibody signal is normalized over a Dam-only control. *Right:* Differential tracks (knockdown – control) highlighting the gain and loss of genome–NL interactions following the depletions of the three topoisomerases. (**B**) LMNB2 pA-DamID signal distribution for control, TOP1, TOP2A, and TOP2B depletion. In control cells, a bimodal distribution usually represents iLAD/LAD partitioning. Results are the average of three independent biological replicates. (**C**) LAD and iLAD score following TOP2B depletion using LMNB1 and LMNB2 mapping data. Results are from two biological replicates and four technical replicates by using two control siRNAs and two TOP2B-specific siRNAs. (**D**) Correlation scatter plots of 20 kb genomic bins for LMNB2 score in control cells (x-axis) and differential LMNB2 score (topoisomerase depletion – control, y-axis) for TOP1, TOP2A, and TOP2B depletions. The blue line represents a linear model; Pearson correlation and *P*-value are shown in the plots. (**E**) Average LMNB1 pA-DamID score around LAD borders for control and TOP2B depleted samples. The effect of three different siRNAs for TOP2B depletion are shown separately. Genome–NL contact levels in control samples are the average of two biological replicates and four technical replicates generated using two control siRNAs. The solid line and the shaded area represent the mean signal and 95% confidence interval of the mean, respectively.

To understand the impact of DNA topoisomerases loss on genome–NL association *in vivo*, we depleted either TOP1, TOP2A, or TOP2B by RNA interference (Supplementary Fig. S1A) and mapped genome–NL interactions using pA-DamID, a recent adaptation of DamID that provides improved temporal resolution [53]. Interestingly, all three DNA topoisomerases could modulate genome–NL interactions to some extent and at specific genomic locations, with both gain and loss of DNA–NL interactions following topoisomerase loss (Fig. 1A). However, we observed a more robust effect of TOP2B depletion. Unlike TOP1 and TOP2A, TOP2B depletion led to a substantial redistribution of genome–NL contacts. This was characterized by a switch from the bimodal distribution typical of control cells to a monomodal distribution in TOP2B-depleted cells (Fig. 1B), implying an overall loss of partitioning of the genome between the NL and the nuclear interior. Gains and losses of genome–NL interactions involved large parts of the genome, and changes could be detected in both LAD and iLAD regions (Fig. 1A).

Following TOP2B depletion, LADs showed a significant reduction in NL association and iLADs often gained NL interactions. This trend was observed by pA-DamID of both LMNB1 and LMNB2 (Fig. 1C and Supplementary Fig. S1B). Such a de-partitioning effect could be adequately described as the slope in a correlation plot of the NL association score in control cells versus changes in NL interactions following topoisomerase depletion (Fig. 1D). This analysis revealed substantially stronger effects for TOP2B loss than for the other two topoisomerases. Although TOP2A knockdown had some effects on genome–NL interactions, we decided not to investigate this enzyme further due to the apparent strong effects on cell cycle progression, consistent with the known role of TOP2A in decatenation and progression through mitosis [78]. In contrast, TOP2B depletion did not alter cell cycle progression (Supplementary Fig. S1C).

#### Changes induced by TOP2B depletion are robust and reproducible

To verify these results, we performed several additional controls. First, we induced TOP2B depletion with two additional siRNAs. All depletion experiments showed similar effects and a high degree of genome-wide correlation, ruling out siRNA-specific effects (Fig. 1E and Supplementary Fig. S1D and E). Second, we considered that the pA-DamID score is a normalized signal calculated as the log_2_(ratio) of two different signals, one from an antibody (e.g. LMNB2) and the other from the use of a freely diffusible Dam. This second signal takes into account potential changes in DNA accessibility or sequence distortion. To rule out the possibility that TOP2B loss causes changes in genome accessibility, we examined the changes in the ‘Dam only’ and ‘LMNB2 only’ datasets after TOP2B knockdown. We found that most of the normalized signal changes were due to changes in LMNB2 signal levels (Supplementary Fig. S2A and B). Third, to rule out a possible normalization problem, we performed calibrated pA-DamID in RPE1 cells (control and TOP2B depleted) spiked in with mouse embryonic fibroblasts (Supplementary Fig. S2C, see the ‘Materials and methods’ section). Data calibrated to mouse reads showed genome-wide profiles almost identical to uncalibrated data for changes in chromatin–NL contacts after TOP2B knockdown (Supplementary Fig. S2D and E). Thus, the changes in genome–NL interaction after TOP2B depletion described here are not an artefact of the normalization process.

#### TOP2B control over genome–NL interactions is conserved in other cell lines

We extended our analysis to additional cell lines by measuring genome–NL interactions in HCT116 and K562 cell lines that are hypomorphic for TOP2B. Both cell lines showed weaker LAD/iLAD partitioning upon TOP2B depletion, although to a lesser extent than in RPE1 cells (Supplementary Fig. S3A–D). Taken together, these data support a role for TOP2B in controlling genome–NL interactions, which is conserved in different cell lines.

#### Catalytic inhibition of TOP2 alters chromatin–NL contacts

Finally, we explored the effects of inhibiting the catalytic activity of TOP2. We treated RPE1 cells for 3 h with two different inhibitors, Merbarone and ICRF193, which block two distinct steps of the TOP2 catalytic cycle [79] and mapped chromatin–NL contacts. Both drugs induced a partial de-partitioning of chromatin–NL interactions, although at a weaker level than TOP2B depletion (Supplementary Fig. S3E). Similar results were seen also in the GM12878 cell line after 10 min of TOP2 inhibition by ICRF193 (Supplementary Fig. S3F). Thus, inhibition of the TOP2 catalytic activity promotes de-partitioning of chromatin–NL contacts, although at a lower level than TOP2B depletion. These results indicate that, at least partially, TOP2 catalytic activity is required to control LAD/iLAD genome partitioning.

### Changes in genome–NL interactions after TOP2B depletion are not explained by changes in transcription

#### Transcription is relatively stable during TOP2B KD

We and others have previously shown that transcriptional activation promotes the physical separation of chromatin from the NL [16–18]. Similarly, transcriptional inhibition favours genome–NL reassociation [18, 19]. Since TOP2B is considered a transcription-associated topoisomerase [80], we asked whether changes in gene expression following TOP2B depletion could explain changes in genome–NL interactions. RNA-seq analysis revealed modest changes in transcription upon TOP2B depletion, with no strong general up or down-regulation in either LADs or iLADs (Supplementary Fig. S4A). In details, 917 genes were significantly upregulated and 645 genes downregulated (Supplementary Fig. S4B). However, the overall disregulation was relatively marginal, as only 219 upregulated and 118 downregulated genes showed >2-fold change, suggesting that TOP2B depletion did not induce dramatic changes in gene expression at the time point analysed. Differentially expressed genes showed significant but very modest changes in the pA-DamID score (Supplementary Fig. S4C). Similarly, genes with a differential DamID score showed significant but marginal changes in expression levels (Supplementary Fig. S4D). These changes could explain only a small fraction of the overall alterations in NL association observed after TOP2B depletion. Thus, it is unlikely that transcriptional changes induced by TOP2B knockdown are the main cause of the pronounced de-partitioning phenotype.

### Differential topological state between LADs and iLADs

#### LADs and iLADs have distinct topological states

**Figure 2.**
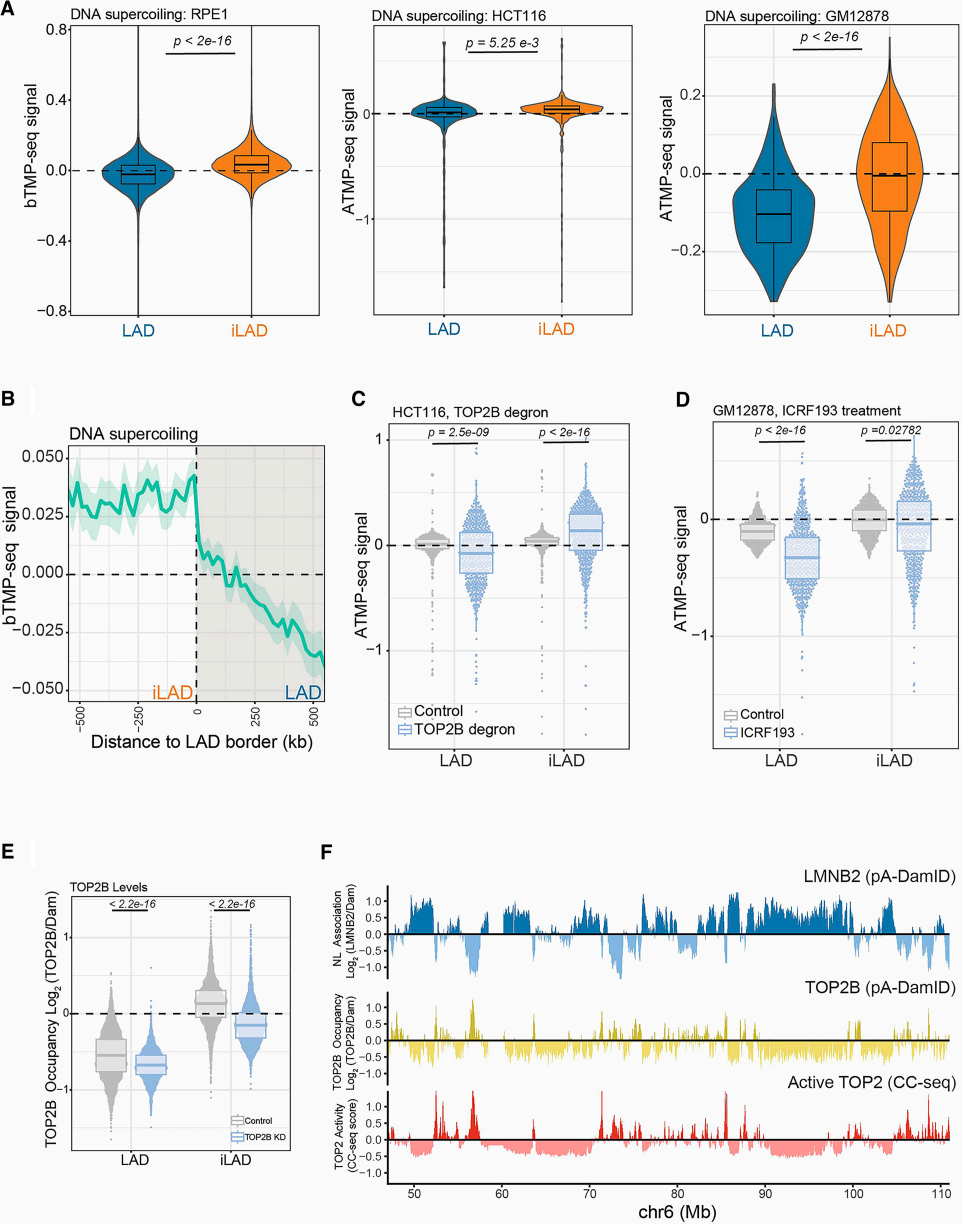
TOP2B controls iLAD and LAD topology. (**A**) *Left panel*: bTMP-seq score [log_2_(IP/input)] for LADs and iLADs in WT RPE1 cells. For visualization purposes only, outliers were removed. *Middle Panel:* ATMP-seq score for LADs and iLADs in HCT116 cells. *Right panel:* ATMP-seq score for LADs and iLADs in GM12878 cells. Results are the average of two independent biological replicates. *P*-values are according to Welch’s *t*-test. ATMP-seq data are from [60]. (**B**) Average bTMP-seq score signal around LAD borders for RPE1 WT cells. The solid line and the shaded area represent the mean signal and 95% confidence interval of the mean, respectively. 500 kb windows inside and outside LADs are shown. (**C**) ATMP-seq score for LADs and iLADs in controls and TOP2B depleted HCT116 cells. (**D**) ATMP-seq score for LADs and iLADs in control and ICRF193 treated GM12878 cells. For panels (C) and (D) results are the average of two independent biological replicates. *P*-values are according to two-sided Wilcoxon’s test. (**E**) TOP2 positioning and activity relative to LADs. TOP2B pA-DamID score in LAD and iLAD in control and TOP2B depleted cells. Results are the average of three independent biological replicates. *P*-values are according to two-sided Wilcoxon’s test. (**F**) Example genomic tracks for pA-DamID signal for LMNB2 and TOP2B and CC-seq. CC-seq data are from [59] and are from three biological replicates.

We then focused on the topological state of LADs in human cells. We used genome-wide maps of DNA supercoiling in RPE1 cells obtained by bTMP-seq, which relies on biotin-psoralen (bTMP) intercalation [57]. Psoralen intercalates more frequently in under-twisted DNA (negatively supercoiled) than in relaxed or positively supercoiled DNA [81]. In wild-type (WT) RPE1 cells, bTMP-seq revealed a clear differential pattern for iLADs and LADs: iLADs show a higher level of psoralen intercalation. In contrast, LADs show a lower underwound state than iLADs (Fig. 2A). Similar results were obtained by analyzing ATMP-seq data in HCT116 and GM12878 cells [60], with minor differences in the levels of the differential supercoiled state of LADs and iLADs among the different cell lines (Fig. 2A). Thus, data from different psoralen-based approaches were consistent and our analysis revealed that LADs are less negatively supercoiled (or more positively supercoiled) than iLADs. Interestingly, on average, LAD borders represented sharp topological transition regions (Fig. 2B).

#### Sequence composition does not bias estimates of LAD topology

We considered that a difference in topological state between LADs and iLADs could be due to sequence bias of psoralen intercalation, given that psoralen has a bias for TA dinucleotides [82]. However, this sequence preference should lead to increased rather than decreased intercalation in LADs, which are generally AT-rich [65]. Furthermore, both in bTMP-seq and ATMP-seq data, the psoralen intercalation signal in naked relaxed DNA is removed from the psoralen intercalation score generated in chromatin, thereby filtering out any potential DNA sequence bias. Therefore, it is unlikely that the differential supercoiling profile of LAD and iLAD is due to psoralen sequence preference.

#### Nucleosome positioning does not bias LAD topology estimates

Nucleosomal occupancy can also reduce psoralen intercalation [83]. Thus, the low levels of bTMP intercalation detected in LADs could be explained by a higher nucleosomal density in these regions. To test this, we measured nucleosomal occupancy for LADs and iLADs using MNase-seq or Strand-seq data for six different cell lines [68–72, 74]. This analysis showed decreased signal in LADs in all the tested cell lines, suggesting that LADs are relatively depleted of nucleosomes (Supplementary Fig. S5A). We observed similar results for MNase data generated in mouse embryonic stem cells (mESCs) [65] (Supplementary Fig. S5B). These results must be considered carefully as the above-mentioned MNase-seq datasets might be biased towards selective disruption of nucleosomes in AT-rich regions like LADs, due to extensive MNase digestion [84]. Thus, we further explored previously reported maps of nucleosome occupancy in mESCs generated by chemical mapping, a technique that should be less biased towards sequence composition [65]. This revealed similar nucleosome density for LADs and iLADs (Supplementary Fig. S5C). However, linker DNA length distributions calculated from chemical mapping data showed that LADs tend to have longer linker DNA than iLADs, suggesting that LADs have a slightly lower nucleosomal density than iLADs (Supplementary Fig. S5D). This result is consistent with data showing that LADs are enriched in histone H1 [85] and that histone H1 binding occurs on longer linker DNA [86]. Thus, lower psoralen intercalation in LADs cannot be explained by increased nucleosomal occupancy and likely reflects a topological feature of heterochromatic and NL-bound regions.

Although we cannot completely rule out that other LAD features affecting chromatin accessibility might affect psoralen intercalation in LADs, these data suggest that LADs may have a different topological state than iLADs, with the former being less underwound than the latter.

### Chromatin relaxation is not sufficient to promote LAD reshaping

#### In vivo chromatin relaxation by break inducers does not alter genome–NL interactions

To probe a possible role of DNA supercoilng in chromatin–NL association we induced dissipation of supercoiling by single-strand break inducers. We mapped genome–NL interactions after 3 h of treatment with two doses of bleomycin. This drug can induce single and double-strand breaks in a dose-dependent manner, promoting the dissipation of dynamic supercoils [57]. At the tested concentrations and time, bleomycin treatment did not affect genome partitioning between the NL and the nuclear interior (Supplementary Fig. S6A). Although we cannot rule out that the lack of effect is due to an unsufficent number of breaks in LADs, these data suggest that simple dissipation of supercoils is not sufficient to promote changes in genome–NL interactions.

#### Ex vivo chromatin relaxation by purified topoisomerases does not alter genome–NL interactions

DNA topoisomerase activity can remove supercoils from chromatin. We performed *ex vivo*chromatin relaxation by treating permeabilized nuclei with purified human type II topoisomerases, either TOP2A or TOP2B. After 30 min of incubation in the TOP2 relaxation buffer in the presence or absence of hTOP2A or hTOP2B, permeabilized nuclei were processed for classical pA-DamID protocol for LMNB2. We detected minor differential genome–NL interactions after incubation with topoisomerase II as compared to incubation with the TOP2 buffer alone (Supplementary Fig. S6B). Although we cannot rule out that exogenous TOP2 proteins were unable to sufficiently access the genomic DNA under these conditions, these results suggest that chromatin relaxation and/or decatenation alone are not sufficient to alter genome–NL interactions *ex vivo*.

### TOP2B controls iLAD and LAD topology independently from chromatin–NL de-partitioning

#### TOP2B regulates DNA supercoiling in LADs and iLADs

To directly estimate the topological changes induced by topoisomerase depletion, we used ATMP-seq data generated in HCT116 cells where TOP2B was rapidly degraded [60]. This analysis revealed that iLADs significantly increase negative supercoiling while LADs tend toward positive supercoil accumulations following TOP2B loss (Fig. 2C). We then analysed the effect of pharmacological inhibition of TOP2 by ICRF193 in GM12878 cells [60]. In this specific condition, LADs accumulate mainly positive supercoiling while iLADs show increased of both positive and negative helical tension (Fig. 2D). The effect in LADs could be explained by the additional inhibition of TOP2A, which was found enriched in these domains [87] and is also targeted by ICRF193, although at a minor level than TOP2B [88]. Thus, type II topoisomerases regulate the differential supercoiling pattern of LADs and iLADs. Intriguingly, TOP2B has a slight preference in removing negative supercoiling from iLADs.

#### Changes in DNA supercoiling induced by TOP2 loss of function do not reflect changes in chromatin–NL contacts

To understand if the topological changes described above reflect changes in chromatin–NL contacts, we compared ATMP-seq data in HCT116 rapidly depleted of TOP2B with changes in chromatin–NL in HCT116 TOP2BKO. This analysis revealed no correlation between these two types of chromatin changes (Supplementary Fig. S6C). Similarly, we could not see any correlation between changes in DNA supercoiling induced by ICRF193 treatment in GM12878 and the corresponding changes in chromatin–NL contacts (Supplementary Fig. S6D). Thus, despite TOP2B being able to control LAD and iLAD topology, the changes in chromatin–NL contacts following TOP2B depletion seem to not depend on supercoiling modulation in interphase.

### TOP2B might protect iLADs from genome–NL association

#### TOP2B positioning and activity are mainly enriched in iLADs

To better understand the relationship between TOP2B and genome–NL association, we focused on TOP2B positioning in the genome. We generated pA-DamID data for TOP2B and validated this in our TOP2B-depleted cells (Fig. 2E). The mapping data showed that TOP2B is relatively depleted in LADs and mainly enriched in iLAD locations, although we could also detect a low TOP2B-specific signal in LADs (Fig. 2E and F, and Supplementary Fig. S7A).

Next, we focused on the localization of catalytically active TOP2B. We used mapping data of catalytically active TOP2 in RPE1 cells generated by CC-seq [59]. This technique detects cleavage complex formation when TOP2 is poisoned on DNA by VP16 treatment, and is a measure of catalytically engaged TOP2. Although CC-seq cannot distinguish between TOP2A and TOP2B, in RPE1 cells most of the CC-seq signal is derived from TOP2B activity [59]. Therefore, this technique can be used as a proxy for TOP2B activity. We found a high positive correlation between our TOP2B pA-DamID data and the CC-seq dataset (Fig. 2F and Supplementary Fig. S7B). Similar to our pA-DamID data, CC-seq showed that LADs are relatively depleted of high TOP2 activity compared to iLADs (Supplementary Fig. S7C). Considering that iLADs tends to show a gain of interactions with the NL upon topoisomerase depletion (Fig. 1C and D) these data might suggest that TOP2B might protect iLADs from association to NL.

### A TOP2B/LBR axis controls genome partitioning between the NL and the nuclear interior

#### Mapping of LBR-associated chromatin

Our data suggest that TOP2B may protect iLADs from interacting with the NL. However, this model does not fully explain why genome–NL interactions in LADs become weaker following TOP2B depletion. Indeed, a competition model would require LADs to be released from the NL. We wondered whether TOP2B could mediate genome–NL interactions in coordination with an NE tether. We focused on the LBR. This protein is an organizer of heterochromatin at the nuclear periphery [89, 90]. Interestingly, *in vitro* data suggest that the chromatin-interacting nucleoplasmic domain of LBR binds DNA with an affinity dependent on DNA curvature and supercoiling [91]. These considerations led us to investigate a possible link between TOP2B and LBR. We first mapped contacts between the genome and LBR utilizing pA-DamID and an LBR-specific antibody. We confirmed the high specificity of the resulting maps, by using an LBR knockout cell line as control (Supplementary Fig. S8A). In wild-type cells, genome–LBR interactions correlated strongly with genome–LMNB2 interactions (Supplementary Fig. S8B) and anti-correlated with TOP2 activity (Supplementary Fig. S8C), in agreement with previous data (Supplementary Fig. S7C). In general, LBR mapping showed a de-partitioning effect similar to mapping of LMNB1 and LMNB2 interactions following TOP2B depletion (Supplementary Fig. S8D), and the changes induced by TOP2B loss on genome–LBR interactions correlated strongly with changes in genome–LMNB1 contacts (Supplementary Fig. S8E).

#### A subset of LADs preferentially lose contact with LBR following TOP2B depletion

**Figure 3.**
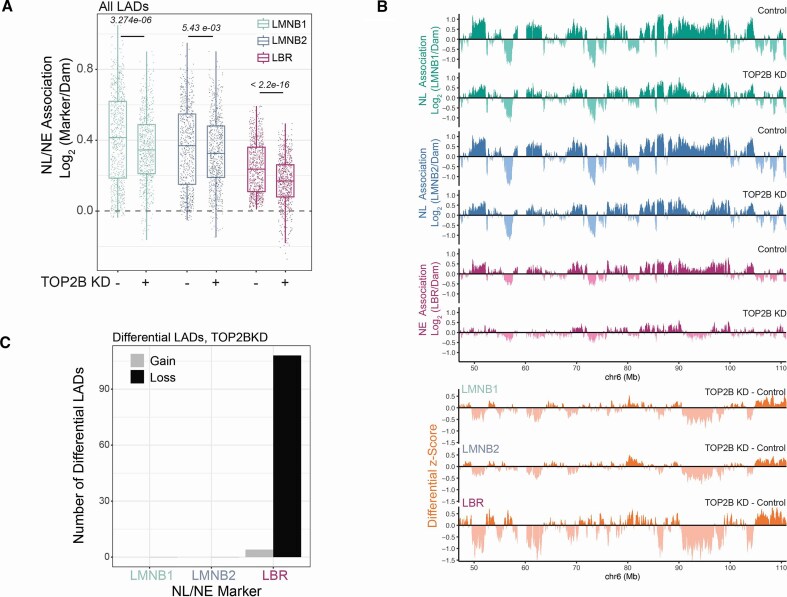
A subset of LADs preferentially loses contacts from LBR following TOP2B loss. (**A**) LAD pA-DamID scores for control and TOP2B depleted cells for three different marks: LMNB1, LMNB2, and LBR. *P*-values are according to Wilcoxon’s test. (**B**) Example genomic tracks of LMNB1, LMNB2 and LBR pA-DamID signal for control and TOP2B depleted RPE1 cells and relative differential tracks (TOP2B knockdown – control) using z-scored signal from the three different antibodies; 20-kb bins were used. One unit of z-score corresponds to 0.297, 0.204, 0.219 in differential pA-DamID score for LMNB1, LMNB2, and LBR, respectively. (**C**) Results of Voom–Limma analysis used to call LADs that significantly gain or lose interaction with LMNB1, LMNB2, and LBR following TOP2B depletion. Results are from two biological replicates and four technical replicates generated using two negative control siRNAs and two TOP2B-specific siRNAs.

A direct comparison of LMNB1, LMNB2, and LBR at the LAD level revealed a more pronounced and significant weakening of interactions of LADs with LBR (Fig. 3A and Supplementary Fig. S8F). This result was particularly striking considering that the LBR signal in RPE1 cells had a significantly lower dynamic range compared to signals generated with LMNB1 and LMNB2 mapping and should therefore be less sensitive in capturing potential differences (Supplementary Fig. S8G). A visual comparison of differential z-scores (TOP2BKD-Control) for the three markers revealed a detachment detectable in LAD regions, significantly stronger for LBR-chromatin contacts (Fig. 3B). Consistent with these observations, Limma–Voom differential analysis [53, 56] identified 108 LADs (out of 765 LADs, 14%) that significantly lose interactions after TOP2B depletion, specifically with LBR, but not with LMNB1 and LMNB2 (Fig. 3C). We conclude that in response to TOP2B loss, a subset of LADs preferentially lose contact with the NE tether LBR.

#### LBR knockout causes substantial changes in genome–NL interactions

To investigate a possible link between TOP2B and LBR, we generated LMNB2 pA-DamID data in RPE1 cells in which we knocked out LBR by CRISPR genome editing (Supplementary Fig. S8H) [20]. In RPE1 the loss of LBR caused a clearly detectable de-partitioning of genome–NL interactions (Supplementary Fig. S8I) and changes in LAD strength, with a subset of strong LADs showing a marked reduction of LMNB2 signal (Supplementary Fig. S8L). Differential LAD calling analysis identified 220 LADs significantly losing interactions with LMNB2. Three hundred LADs increased their NL association upon LBR loss (Supplementary Fig. S8M), a phenomenon we did not observe upon TOP2B depletion. This result probably reflects compensatory movements induced by the prolonged absence of LBR.

#### TOP2B knockdown mirrors the effects of LBR knockout

**Figure 4.**
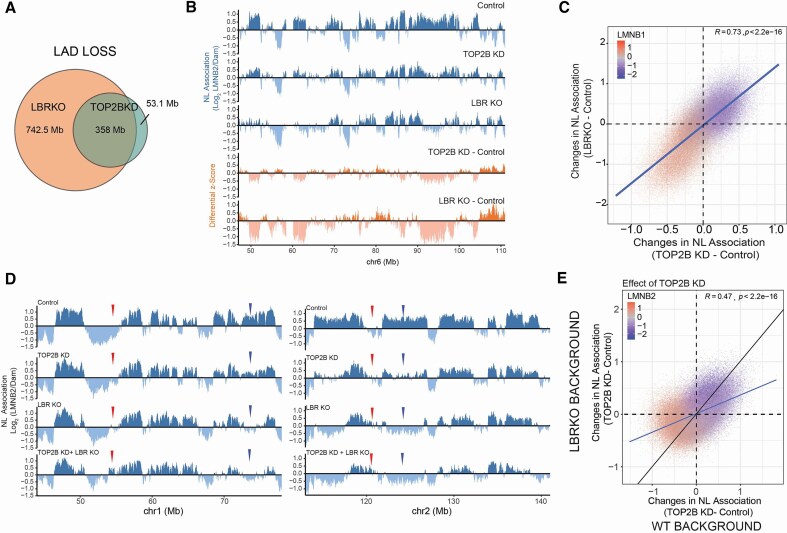
A TOP2B/LBR axis controls genome partitioning between the NL and the nuclear interior. (**A**) Genomic overlap (in Mb) for LADs released from LBR during TOP2B depletion and LADs that significantly lose genome–NL interactions following LBR Knockout. (**B**) *Blue tracks:* example genomic tracks of LMNB2 pA-DamID for the control sample, TOP2B knockdown, and LBR knockout; 20-kb bins were used. *Orange tracks:* differential z-scored LMNB2 tracks (depletion-control) for TOP2B and LBR depletions. (**C**) Correlation scatter plot for differential LMNB2 pA-DamID scores for TOP2B depletion (TOP2B knockdown – control, x-axis) and LBR knockout (y-axis LBR knockout – control, y-axis). The results are an average of four biological replicates for LBR knockout. For TOP2B depletion results are from two biological replicates and four technical replicates by using two different control siRNAs and two different TOP2B-specific siRNAs. (**D**) Effects of co-depletion of TOP2B and LBR on genome–NL interactions: Two example genomic tracks of LMNB2 pA-DamID for the control sample, TOP2B knockdown, LBR knockout, and co-depletion of TOP2B and LBR; 20-kb bins were used. The antibody signal is normalized over a Dam-only control. *Red arrows*: Regions that progressively switch from iLAD to LAD state during the different depletions. *Blue arrows*: Regions that progressively switch from LAD to iLAD state during the different depletions. (**E**) Correlation between changes in genome–NL interactions induced by depletion of TOP2B in WT and LBR knockout background. Results are from four biological replicates. The blue line represents a linear model; Pearson correlation and *P*-value are shown in the plots.

A direct comparison with regions that lose contact with LBR upon TOP2B depletion revealed that approximately 87% of LADs that significantly detach from LBR following TOP2B knockdown also undergo NL detachment upon LBR knockout (Fig. 4A). Similarly, almost half of the LADs that significantly lose interaction with the NL in LBR knockout cells also detach from LBR upon TOP2B depletion. These data suggest that TOP2B and LBR may modulate genome–NL contacts in a similar manner. To test this directly, we compared the two differential scores (perturbation – control) for TOP2B knockdown and LBR knockout. We found a striking correlation between changes in genome–NL contacts after TOP2B depletion and after LBR knockout (Fig. 4B and C). The magnitude of the effect was greater for LBR knockout than for TOP2B depletion. These data show that TOP2B and LBR control the interactions between the genome and the NL in very similar ways. It is also important to note that loss of TOP2B did not induce changes in either transcript [log_2_(fold change) = −0.21, p adj = 0.54] or protein levels for LBR (Supplementary Fig. S8H).

#### Co-depletion points to a sub-additive role for TOP2B and LBR

To better dissect the role of TOP2B and LBR in regulating genome–NL interactions, we depleted TOP2B in the LBR knockout background (Supplementary Fig. S8H). In the absence of LBR, depletion of TOP2B caused further de-partitioning (Fig. 4D), mostly of the same genomic regions as it controlled in the presence of LBR (Fig. 4E). However, we observed stronger changes in the interaction with the NL in the presence rather than in the absence of LBR, as shown by a flattening of the correlation line between the differential scores in the two different genetic backgrounds (Fig. 4E). Thus, TOP2B and LBR are neither epistatic nor synergistic in their effects on NL interactions, but show sub-additive phenotypes.

### Co-depletion of TOP2B and LBR recapitulates LAD reshaping typical of OIS

#### TOP2B and LBR depletion mimics changes typical of OIS

**Figure 5.**
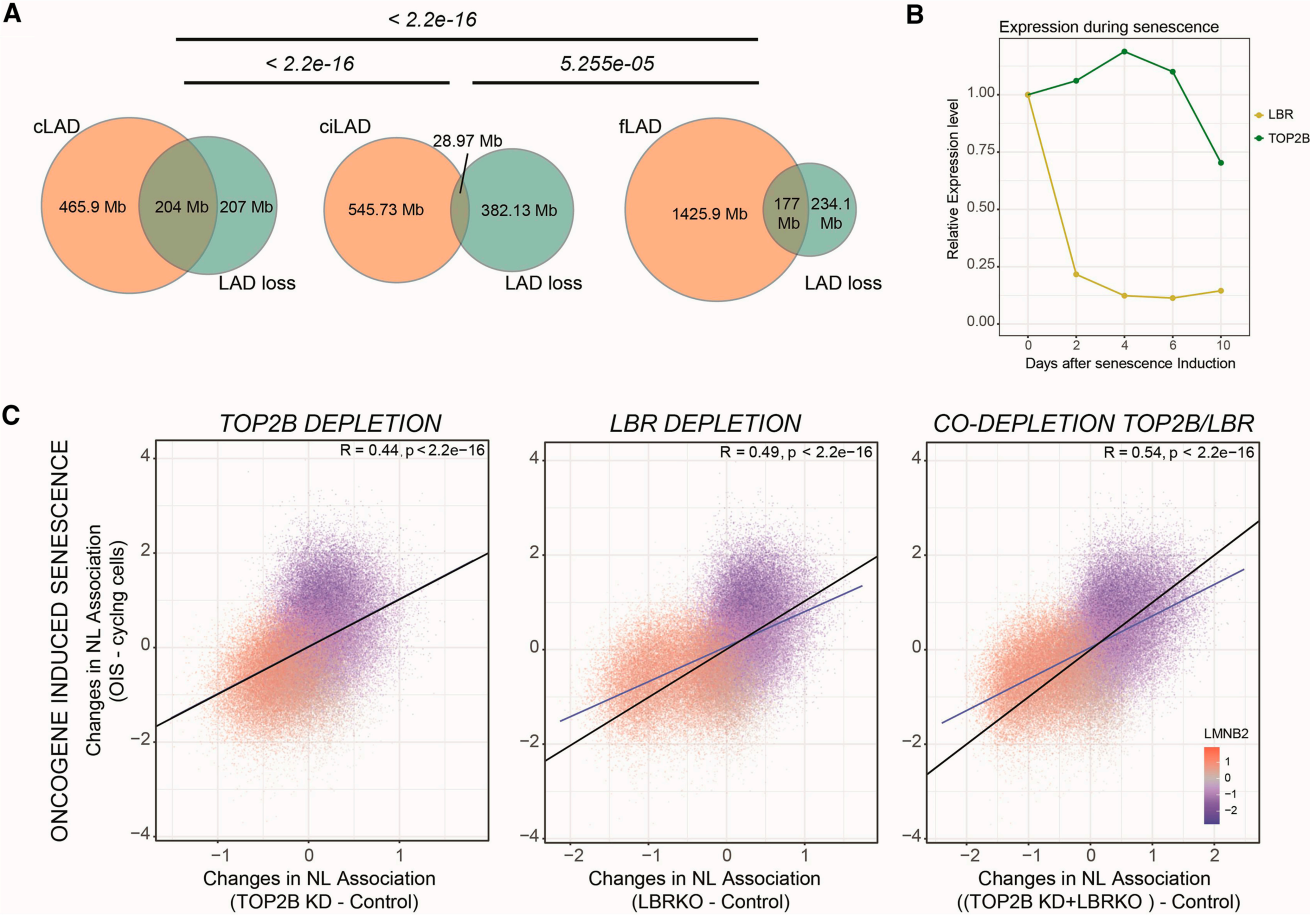
TOP2B and LBR co-depletion mimics LAD reshaping typical of OIS. (**A**) Genomic overlap in Mb for LADs released by LBR during TOP2B depletion and cLADs, fLADs, and ciLADs. Statistical significance was calculated with a Fisher’s test. (**B**) RNA-seq data showing transcript level for LBR and TOP2B during OIS. Results are from [76] and are the average of three independent biological replicates. (**C**) Correlation scatter plot of 20-kb genomic bins for differential LMNB2 scores (x-axis) for TOP2B knockdown (left plot), LBR knockout (middle plot), and co-depletion of TOP2B and LBR (left plot) and changes in lamina association after OIS (y-axis). The blue line represents a linear model; Pearson correlation and *P*-value are shown in the plots. Data in RPE1 cells are the average of four biological replicates. OIS DamID data are from [75] and are the average of three independent biological replicates. Since OIS data were generated in Tig3 fibroblasts and not RPE1 cells, discordant bins in control data between the two cell lines were filtered out to allow direct comparisons of conserved genome–NL interactions. This led to the removal of near 30% of the total number of 20-kb bins.

The co-depletion of TOP2B and LBR showed the most substantial de-partitioning effect (Supplementary Fig. S9A) with genomic regions switching from LAD to iLAD state and vice versa (Fig. 4D). This relatively strong phenotype reminded us of striking LAD reshaping events typical of OIS. During this type of senescence, cLADs, defined as LADs conserved among different cell lines, detached from the NL [75, 92]. We wondered if LADs detaching after TOP2B depletion were predominantly cLADs. Using a LAD atlas that identified constitutive and facultative LADs [26] (fLADs, LADs that are not maintained across different cell lines), we found an overlap between lost LADs induced by TOP2B knockdown and cLADs, significantly higher than the overlap with fLADs or with ciLADs (Fig. 5A). Thus, similarly to what happens during OIS, cLADs become weaker following TOP2B depletion.

This prompted us to examine the expression of TOP2B and LBR during OIS using published RNA-seq data [76]. Interestingly, while LBR expression is downregulated during OIS, TOP2B expression remains relatively high, suggesting that these two proteins are differentially regulated during OIS (Fig. 5B). To further explore a possible link between TOP2B, LBR, and senescence, we directly compared our depletion experiments (TOP2B knockdown, LBR knockout, and co-depletion) with DamID data generated in OIS cells [75]. We found positive correlations between changes in genome–NL interactions during all perturbations and those typical of OIS. The correlation was particularly robust when we compared OIS with co-depletion of TOP2B and LBR (Fig. 5C). Cell cycle analysis revealed no alteration in cell cycle progression for either single or double depletions (Supplementary Figs S1C and S9B). In line with these data, TOP2B depleted cells did not show evidence of activated SASP (senescence-associated secretory phenotype) response, a phenotype typical of senescent cells [93] (Supplementary Fig. S9C). Thus, despite mimicking the LAD remodelling typical of OIS, single and co-depletion of TOP2B and LBR did not induce senescence in our model.

### Heterochromatin is stable in detached LADs during TOP2B depletion

#### LAD heterochromatin marked by H3K9me3 is repositioned after TOP2B loss

**Figure 6.**
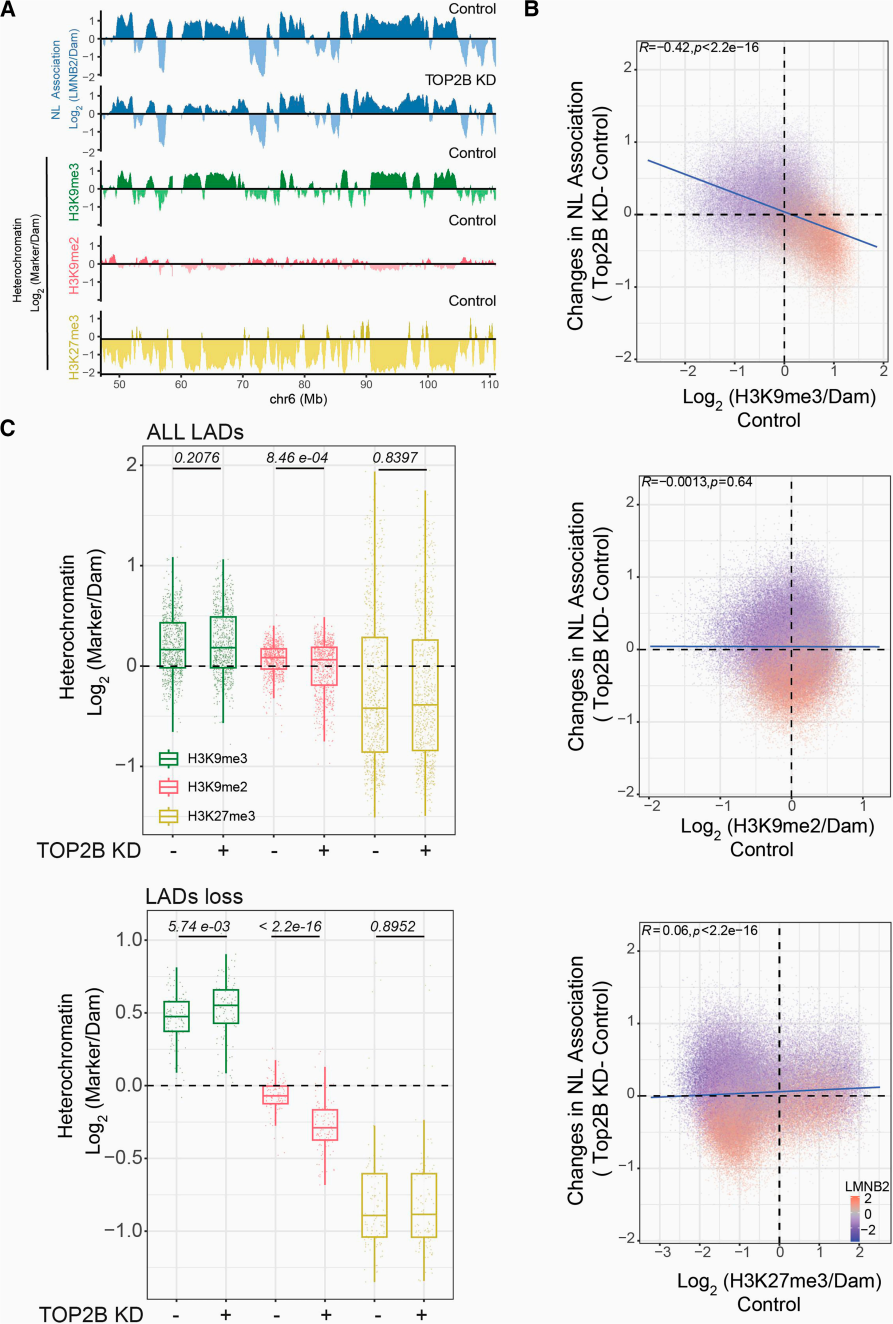
Stability of heterochromatin in LADs released by LBR after TOP2 loss. (**A**) Example genomic track for pA-DamID signal for LMNB2 (control and TOP2BKD cells), H3K9me3 (control cells), H3K9me2 (control cells), and H3K27me3 (control cells); 20-kb bins were used. The antibody signal is normalized over a Dam-only control. (**B**) *H3K9me3-marked heterochromatin detaches from the NL during TOP2BKD*. Correlation scatter plot of 20-kb genomic bins for H3K9me3, H3K9me2, and H3K27me3 scores in control cells (x-axis) and differential LMNB2 score (TOP2BKD – control, y-axis). The blue line represents a linear model; Pearson correlation and *P*-value are shown in the plot. (**C**) H3K9me3, H3K9me2, and H3K27me3 scores in control and TOP2B depleted cells in all LADs (top panel) and LADs released by LBR after TOP2B knockdown (bottom panel). Results are from two independent biological replicates. *P*-values are according to Wilcoxon’s test.

**Figure 7.**
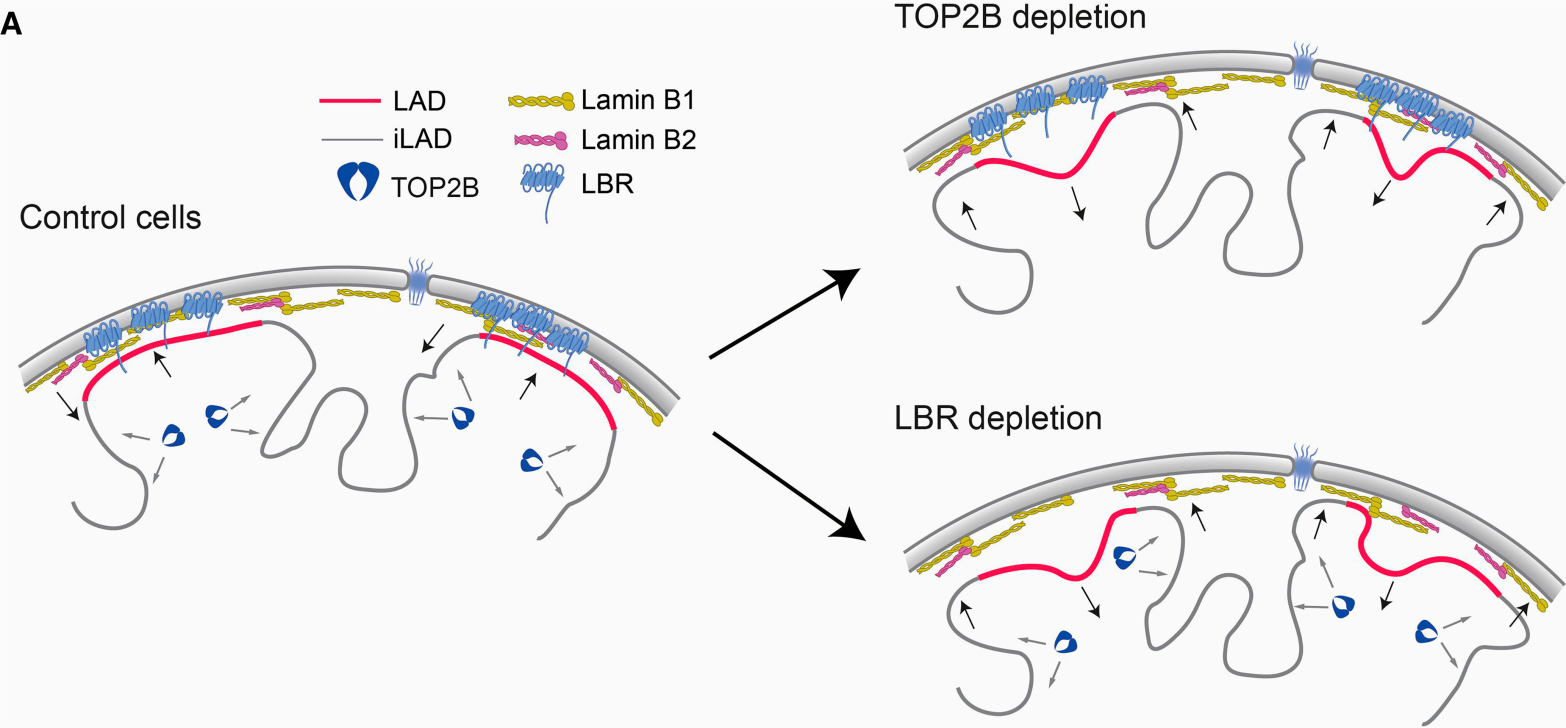
Possible model on how TOP2B and LBR can reinforce LAD/iLAD partitioning: LBR tethers LADs at the nuclear periphery while TOP2B maintain iLAD away from the NL.

LADs are heterochromatic regions frequently marked by H3K9 and H3K27 methylation methylation [1, 4, 5], and LBR is well known as a tether that anchors heterochromatin at the NE [13, 14, 89]. In addition, data suggest that TOP2 can regulate heterochromatin in both yeast and mammals [94, 95]. We investigated whether heterochromatin in LADs is stable following loss of TOP2B and partial release from LBR by using pA-DamID to map H3K9me3, H3K9me2 and H3K27me3 genome-wide. In control RPE1 cells, H3K9me3 was enriched in the central part of LADs. H3K9me2 and H3K27me3 marked the distal part of LAD regions as previously described [96, 97] (Supplementary Fig. S10A), and were relatively less abundant in the part of LADs where H3K9me3 was present. Notably, changes in genome–NL interactions after TOP2B depletion were negatively correlated with H3K9me3, but not with H3K9me2 and H3K27me3 (Fig. 6A and 7B). This result suggests that strong LADs that detach from the NL after TOP2BKD are enriched in H3K9me3 and point to a mechanism where this type of heterochromatin is released from LBR after TOP2B depletion. Thus, in RPE1 cells, TOP2B controls the distribution of H3K9me3-marked heterochromatin at the NE.

#### Stability of H3K9me3 following TOP2B loss

We then measured the levels of H3K9me3, H3K9me2, and H3K27me3 after TOP2B depletion. We calculated a heterochromatin score of the three marks for LADs released from LBR following TOP2B knockdown and for the ensemble of all LADs as a control. The data show that the levels of H3K9me3 increased slightly in regions released from LBR following TOP2B loss. Thus, H3K9me3 is not lost following TOP2B depletion, but rather slightly enhanced following release from LBR (Fig. 6C and Supplementary Fig. S10B). These dynamics may reflect a compensatory mechanism triggered by release from the NE. In agreement with this result, genes in LADs that lose LBR contacts do not show any transcriptional change (Supplementary Fig. S10C).

#### H3K9me2 is sensitive to TOP2B loss

We noticed that H3K9me2 showed a peculiar behaviour. Although this mark is relatively low in LADs that lose contact with LBR, its modulation after TOP2B KD follows the changes in DNA–NL association. LADs that preferentially lost interactions with LBR lost even more H3K9me2 (Fig. 6C and Supplementary Fig. S10B). We propose that H3K9me2 is sensitive to the NL environment. However, given the low levels of this mark in these regions, it is unlikely that these changes are the drivers of the genome–NL interactions that are reshaped during TOP2B depletion. Thus, these data show that following TOP2B depletion, H3K9me3-marked heterochromatin released from LBR is not lost.

## Discussion

This study reveals a role for TOP2B in regulating chromatin contacts with the NL. Upon TOP2B depletion, cLADs marked by H3K9me3 preferentially detach from the NL, whereas iLADs show increased NL interactions. Importantly, H3K9me3 itself remains largely unaffected. These data suggest a mechanism by which heterochromatin is not disrupted during TOP2B knockdown and is instead repositioned away from the NL. These changes in NL interactions are remarkably similar to those observed during OIS [75] (Fig. 5).

### A competition model of chromatin–NL interactions

Several lines of data in *C. elegans* and humans have suggested a competition model for genome partitioning between the NL and the nuclear interior. This model proposes that in a single nucleus, the NL has a limited capacity to interact with the genome [1, 18, 25, 26, 98]. We found that TOP2B is mainly concentrated in iLAD regions, and that some of these regions gain interactions with the NL following topoisomerase depletion. These data suggest that TOP2B may protect iLADs from association with the NL. If TOP2B is limiting, iLADs might interact more frequently with the NL and compete with LADs for NL association. This phenomenon could be enhanced by the concomitant release of LADs when LBR is simultaneously depleted.

### A TOP2B/LBR axis to partition the genome between the NL and the nuclear interior

We propose that TOP2B and LBR may help to compartmentalize the genome between the nuclear periphery and the nuclear interior. Mapping data show that these two proteins occupy two distinct genomic compartments in interphase: LBR interacts with LADs and TOP2B binds to iLADs. Our co-depletion experiments show that TOP2B depletion can still trigger changes in genome–NL interactions even without LBR, suggesting that these two proteins are not epistatic. We propose that these two proteins form an axis that allows correct genome segregation between the NL and the interior by acting on two opposite parts of the genome (Fig. 7A). The striking similarity in the way LBR and TOP2B shape genome–NL interactions could suggest that the activity of these two proteins is linked and coordinated. This hypothesis is supported by the more pronounced dissociation of LAD chromatin from LBR than from lamins upon loss of TOP2B.

### Possible mechanisms of action of TOP2B

Our analysis of DNA supercoiling suggests that although TOP2B regulates DNA supercoiling in LADs and iLADs in asynchronous cells, topological changes induced by TOP2B loss correlate poorly with chromatin–NL contact changes. Thus, DNA supercoiling modulation in interphase is unlikely to impact chromatin–NL contacts directly. The use of TOP2 catalytic inhibitors shows a modest effect on chromatin–NL interactions, suggesting that the catalytic activity of TOP2 might be, at least partially, involved in this process. However, our results indicate that TOP2B depletion by RNA interference has a more substantial effect than inhibitors. It is possible that long-term TOP2B loss of catalytic function might be required to alter genome–NL interactions. Passage through mitosis might be crucial, and TOP2B could work at the mitotic exit when most chromatin–NE/NL interactions are re-established. TOP2B might help LBR to bind chromatin at the mitotic exit, and the two proteins then segregate into two distinct genome compartments in interphase. In this model, both a transient modulation of DNA supercoiling or the regulation of decatenation at the mitotic exit [42] could be important to create a topological substrate optimal for LBR binding. Another possible explanation for the more substantial effect of TOP2B depletion over catalytic inhibition is that TOP2B can also control chromatin–NL interactions independently from its catalytic activity. DNA topoisomerases can work as adaptor, interactor, or scaffold proteins [47, 99–102]. According to this function, TOP2B might work as a structural protein by specifying and protecting iLADs from re-association to NL either in interphase or at mitotic exit. Further studies will be required to understand which peculiar function of TOP2B is necessary for proper genome partitioning between the NL and the nuclear interior.

While this manuscript was under revision, a study focused on chromatin interactions regulated by type II topoisomerases showed how TOP2-dependent changes in higher-order chromatin folding were accompanied by remodeling of chromatin–NL interactions [103]. Thus, novel roles of TOP2 enzymes at the level of chromatin associated with the NL are emerging, highlighting the multifunctionality of type II topoisomerases in shaping the 3D genome and the complexity of chromatin–NL interactions.

## Supplementary Material

gkaf964_Supplemental_File

## Data Availability

RNAseq and pA-DamID data generated in this work are available on GEO (accession code GSE277503, https://www.ncbi.nlm.nih.gov/geo/query/acc.cgi?acc=GSE277503). Labnotes and R scripts regarding this study can be found at https://osf.io/sp9ke.
