# Supplementary material for: Coordinated control of genome–nuclear lamina interactions by topoisomerase 2B and lamin B receptor
Source: Nucleic Acids Res. 2025 Sep 26;53(18):gkaf964. doi: 10.1093/nar/gkaf964 (PMC12464825; doi:10.1093/nar/gkaf964)
Supplement: gkaf964_Supplemental_File [file gkaf964_supplemental_file.pdf]

Coordinated control of genome-nuclear lamina interactions by Topoisomerase 2B and Lamin B receptor

Tom van Schaik[1,4,†], Mikhail Magnitov[1], Marcel de Haas[1,2,4], Jeremie Breda[1,4], Elzo de Wit[1], Anna G Manjon[3], René H Medema[3,5], Henrike Johanna Gothe[6], Vassilis Roukos[6,7], Adam J Buckle[8], Catherine Naughton[8], Nick Gilbert[8], Bas van Steensel[1,2,4], Stefano G Manzo[1,4,9*†]

SUPPLEMENTARY DATA

SUPPLEMENTARY FIGUREs

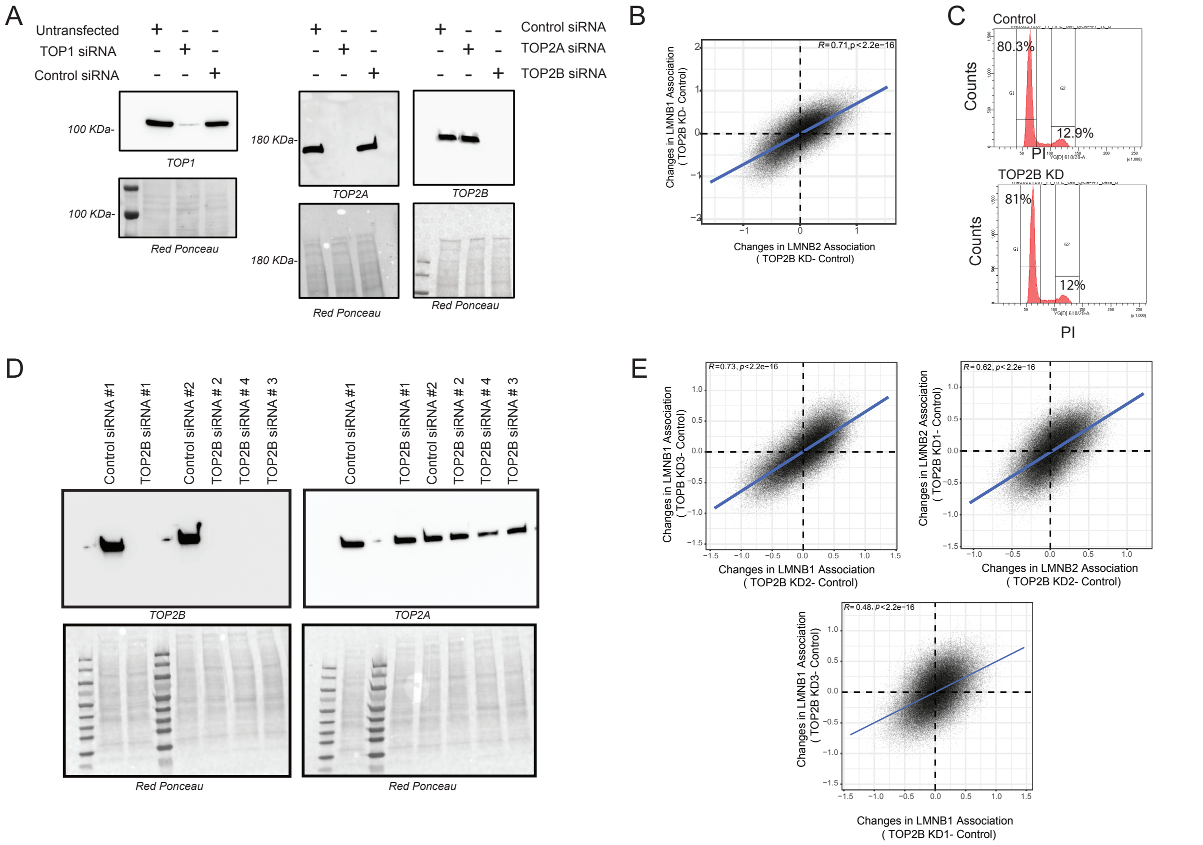

Supplementary Figure 1. Control of TOP2B knockdowns by different TOP2B siRNA and relative effects on chromatin-NL interactions. A) Western blot analysis to measure protein levels for TOP1, TOP2A, and TOP2B following siRNA-mediated depletions. Red Ponceau staining is used as a loading control. B) Correlation scatter plot of 20-kb genomic bins for differential LMNB2 and LMNB1 scores for TOP2B depletion (TOP2B knockdown - control). Results are from the average of two biological replicates. The blue line represents a linear model. Pearson correlation and *P* value are shown in the plot. C) Cell cycle analysis for Control and TOP2B depleted cells, by PI staining. D) Western blot analysis to detect TOP2B and TOP2A levels using different TOP2B siRNAs. Red ponceau was used as loading control. siRNA #4 was not used for further studies as it partially targets TOP2A. E) Correlation scatter plots of 20 kb genomic bins for differential LMNB2 and LMNB1 scores for TOP2B depletion (TOP2B knockdown - control) for three different siRNAs. Results are from two biological replicates. The blue line represents a linear model. Pearson correlation and *P* value are shown in the plot.

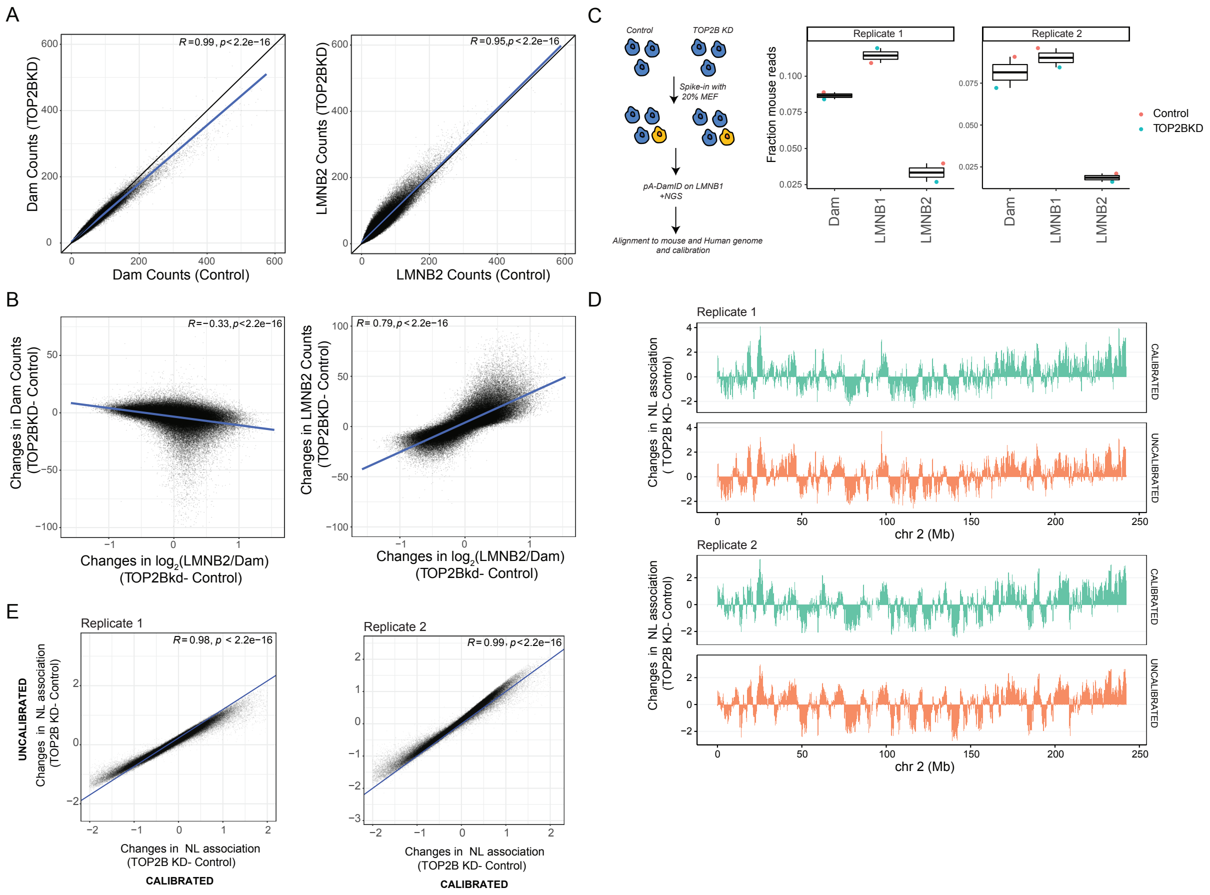

Supplementary Figure 2. **Controls of pA-DamID procedure.** A) Correlation scatter plot for the "Dam only" signal (*left panel*) and "LMNB2 only" signal (*right panel*) for Control and TOP2B depleted cells. B) Correlation scatter plot for differential normalized log_2 LMNB2/Dam score and differential Dam “only” or LMNB2 “only” for Control and TOP2B depleted cells. Data are from three biological replicates. C) *Left:* scheme explaining how calibration with mouse reads was performed. *Right:* Fraction of mouse reads in each sample (Dam, LMNB1, LMNB2) for each replicate. Note: LMNB2 antibody poorly performed in mouse cells and cannot be used for calibrated chromatin-NL contact maps. D) Differential LMNB2 score tracks (TOP2B depletion-control) for calibrated and uncalibrated data for each replicate. E) Genome-wide correlation of differential LMNB2 score (TOP2B depletion-control) for calibrated and uncalibrated data for each replicate. For all correlation scatter plots: the blue line represents a linear model; the black line represents the diagonal; Pearson correlation and *P* value are shown in the plots.

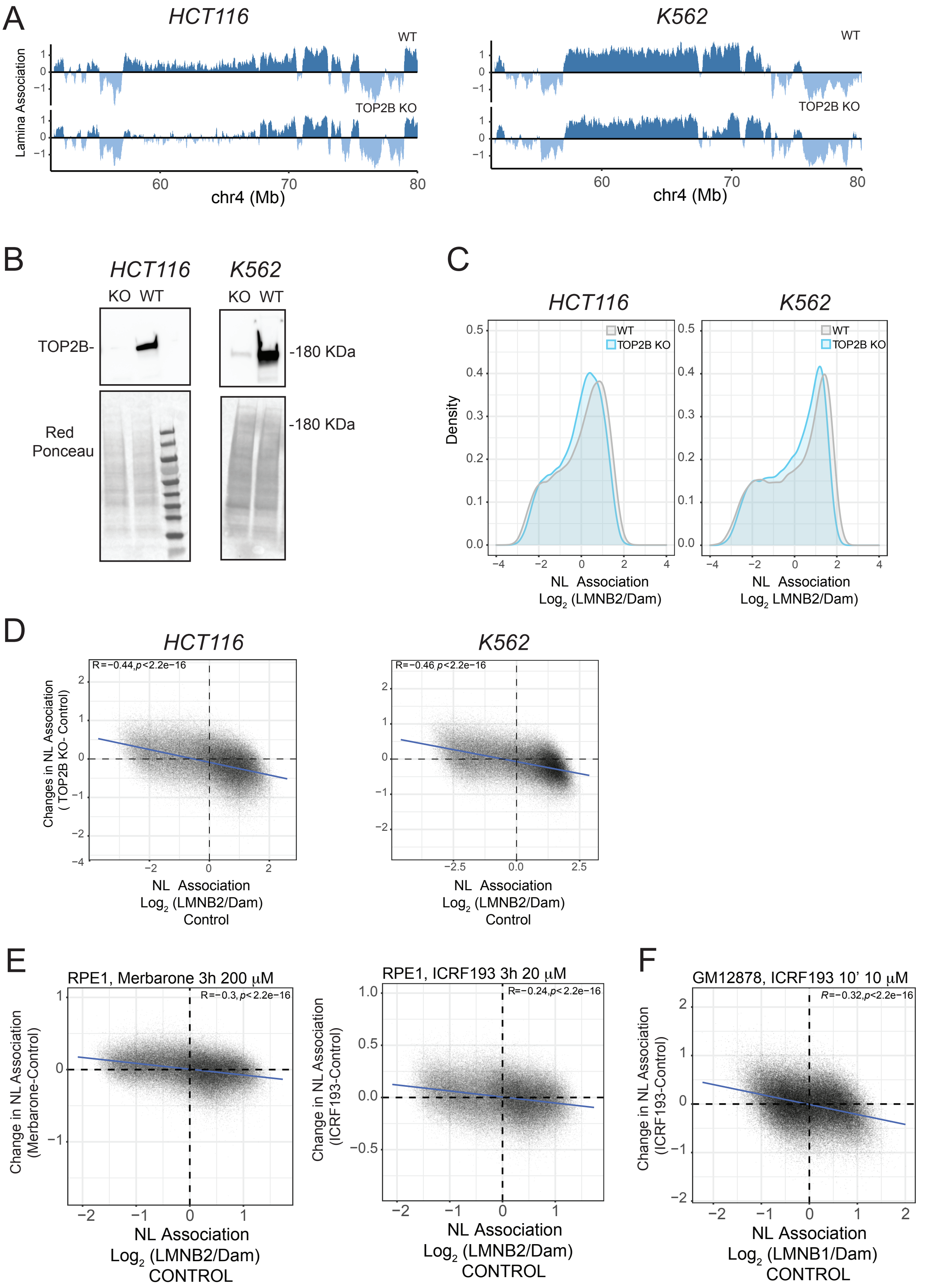

Supplementary Figure 3. TOP2B control over genome-NL contact in HCT116 and K562 cell lines and effect of TOP2 targeting by catalytic inhibitors. A) Example genomic tracks of LMNB2 pA-DamID for control and TOP2B knockout in HCT116 (*left*) and K5632 (*right*) cells. 20-kb bins were used.

B) Western blot showing levels of TOP2B for the four different cell lines. Red ponceau staining was used as a loading control. C) LMNB2 signal distribution for control, and TOP2B knockout HCT116 cells (*left*) and K562 (*right*). D) Correlation scatters plot of 20 kb genomic bins for LMNB2 score in control cells (x-axis) and differential LMNB2 score (TOP2BKO - control, y-axis) in HCT116 (*left*) and K562 (*right*). The results are average of three biological replicates for HCT116 and two biological replicates for K562. E) Correlation scatters plot of 20 kb genomic bins for LMNB2 score in control cells (x-axis) and differential LMNB2 score (Drug - control, y-axis) in RPE1 cells treated with Merbarone (left) or ICRF-193 (right) for three hours at the indicated concentrations. F) Same as E, but for GM12878 cells treated with ICRF-193 for 10' minutes at the indicated concentration and by using a LMNB1 antibody. For E and F, the results are average of two biological replicates. For all correlation scatter plots: the blue line represents a linear model. Pearson correlation and *P* value are shown in the plots.

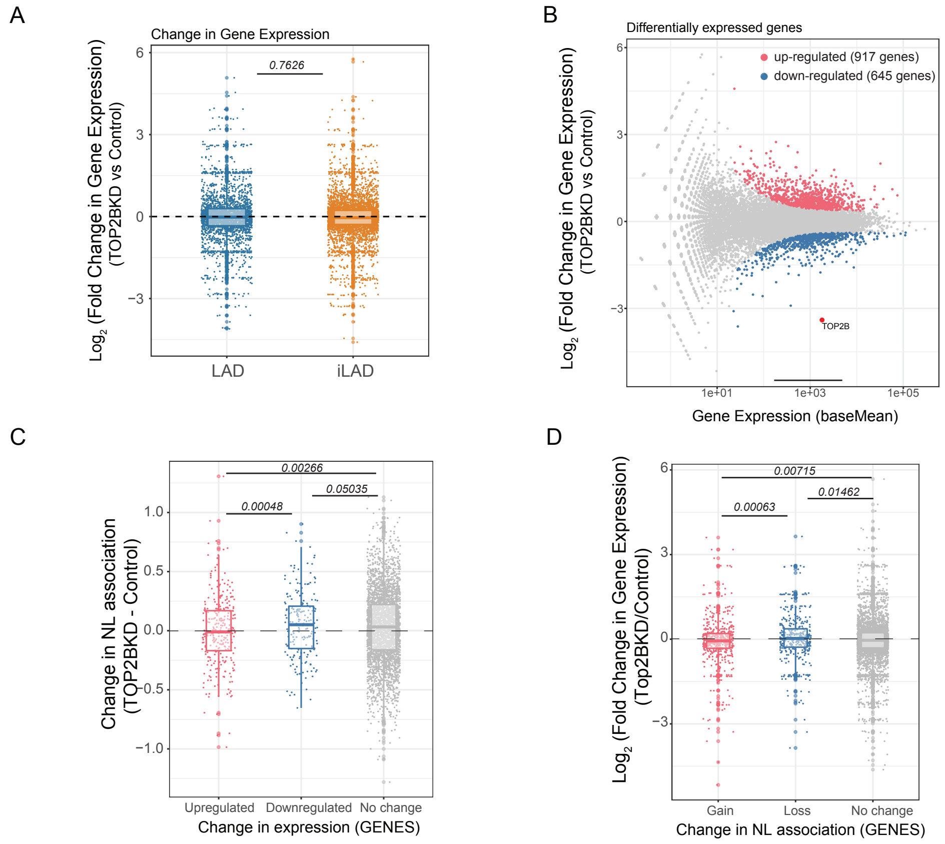

Supplementary Figure 4. **Effects of TOP2B depletion on gene expression.** A) Changes in gene expression following TOP2B depletion for all genes residing in LADs (blue) or iLADs (orange)**.** *P* value is according to two-sided Wilcoxon's test**.** B) MA plot showing differentially expressed genes after TOP2B knockdown. Significant (*p adj* < 0.05) up and down-regulated genes are shown in red and blue respectively. TOP2B gene is highlited. C) Boxplot of differences in LMNB2 interaction score for up-regulated (red), down-regulated (blue), and unchanged genes (grey). D) Boxplot of $\log_2$(Fold Change) in gene expression for differentially attached (red), detached (blue) or stable genes (grey) following TOP2B depletion. To call differentially attached and detached genes a cut-off of +/- 0.4 in differential LMNB2 pA-DamID score was applied. For C and D, *P* values are according to two-sided Wilcoxon's test. Results are the average of two independent biological replicates.

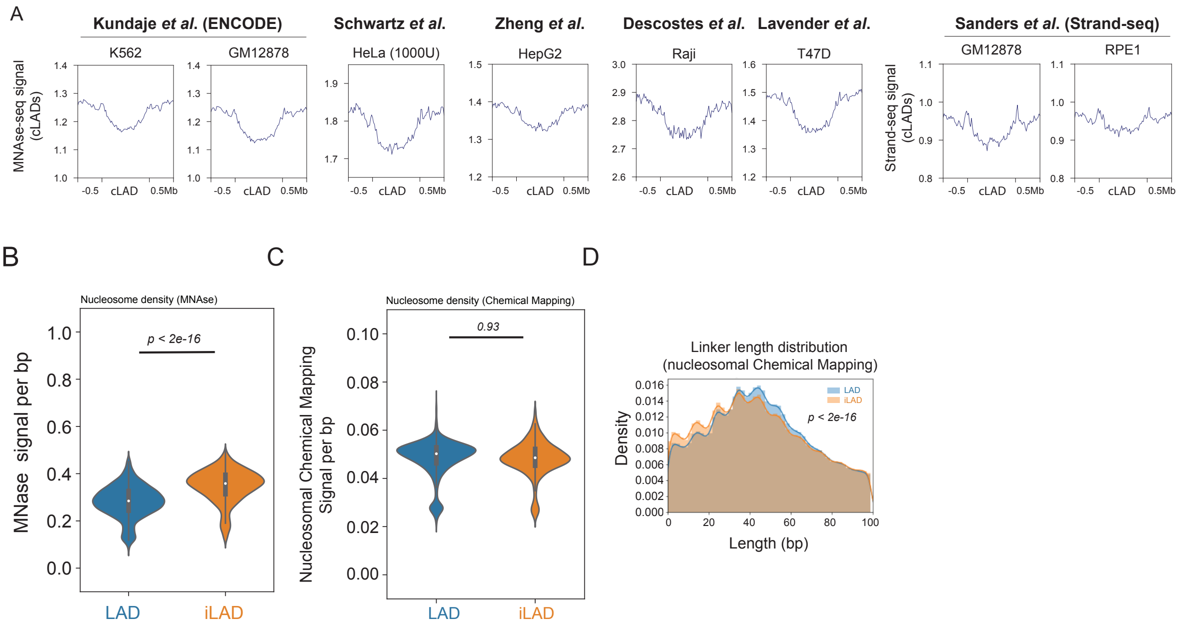

Supplementary Figure 5. Nucleosome mapping in LADs. A) Average profile at constitutive LADs for MNase-seq signal in K562, GM12878, Hela, HepG2, Raji, and T47D cells and Strand-seq signal in GM12878 and RPE1 cells. LADs were scaled to the same length and the 500 kb upstream and downstream of LAD borders are shown. Data are from (1-7). Analysis was performed on cLADs and ciLADs identified in (8). B) MNase signal per bp for LAD and iLAD in mESC. C) Nucleosome signal per base pair for LADs and iLADs in mESC calculated using chemical mapping of nucleosomes. Outlier values from top 0.5 and bottom 0.5 percentiles were excluded from plotting. For B and C, *P* values are according to Welch's t-Test. Analysis was performed on cLADs and ciLADs identified in (9). D) DNA linker length distribution from chemical mapping data for LADs and iLADs in mESCs. Results are from the average of two independent biological replicates. For D, P value is according to Kolmogorov-Smirnov test. For B, C, D, data are from (10)

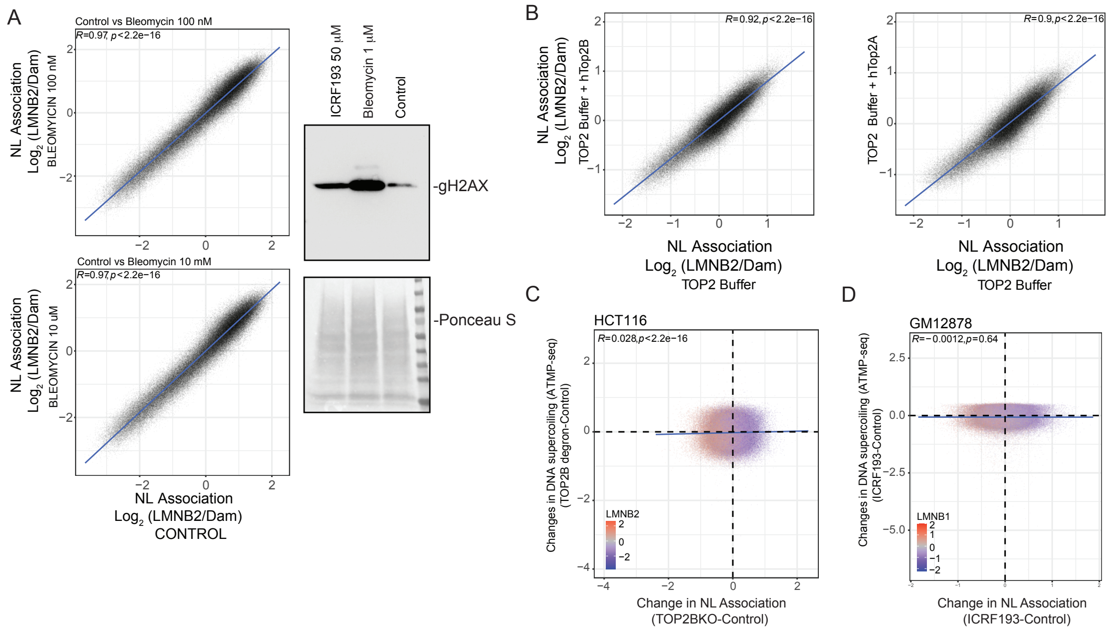

Supplementary Figure 6. Perturbation of dynamic supercoiling does not correlate with genome-NL interactions changes. A) *Left:* Correlation for LMNB2 signal between Control and bleomycin treatments at two different concentrations of bleomycin. *Right*: Western blot showing levels of γH2AX following three hours of treatment with bleomycin or ICRF193 at the indicated concentrations. Red ponceau staining was used as a loading control. B) Correlation for LMNB2 pA-DamID signal for control RPE1 permeabilized and resuspended in TOP2 Buffer (x-axis) or TOP2 Buffer plus human purified TOP2A (*right*) or TOP2B (*left*), (y-axis). 20-kb bins were used for the analysis. The antibody signal is normalized over a Dam-only control. C) Correlation between changes in chromatin-NL contacts (pA-DamID, x-axis) and DNA supercoiling (ATMP-seq, y-axis) during TOP2B depletion in HCT116 cells. D) Correlation between changes in chromatin-NL contacts (pA-DamID, x-axis) and DNA supercoiling (ATMP-seq, y-axis) during TOP2B inhibition by ICRF193 in GM12878 cells. ATMP-seq data are from (11). For all correlation scatter plots: the blue line represents a linear model. Pearson correlation and *P* value are shown in the plots.

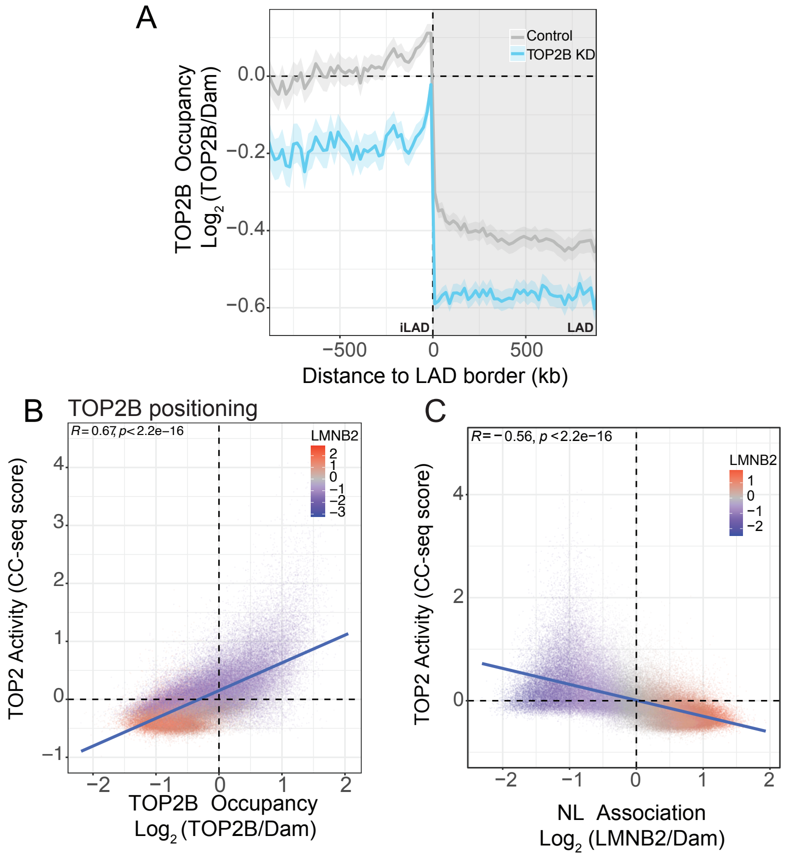

Supplementary Figure 7. Genome wide-mapping of TOP2B positioning and activity A) Average pA-DamID scores for TOP2B around LAD borders for RPE1 cells control (grey) and depleted for TOP2B (blue). The solid line and the shaded area represent the mean signal and 95% confidence interval of the mean, respectively. B) Correlation scatter plot between mapping data for TOP2B generated by pA-DamID and CC-seq signal that measure catalytically active TOP2. C). Correlation scatter plot between CC-seq signal and NL association measured by LMNB2-pA-DAmID. For all correlation scatter plots: the blue line represents a linear model; the black line represents the diagonal; Pearson correlation and *P* value are shown in the plot. Results are from three biological replicates. CC-seq data are from (12).

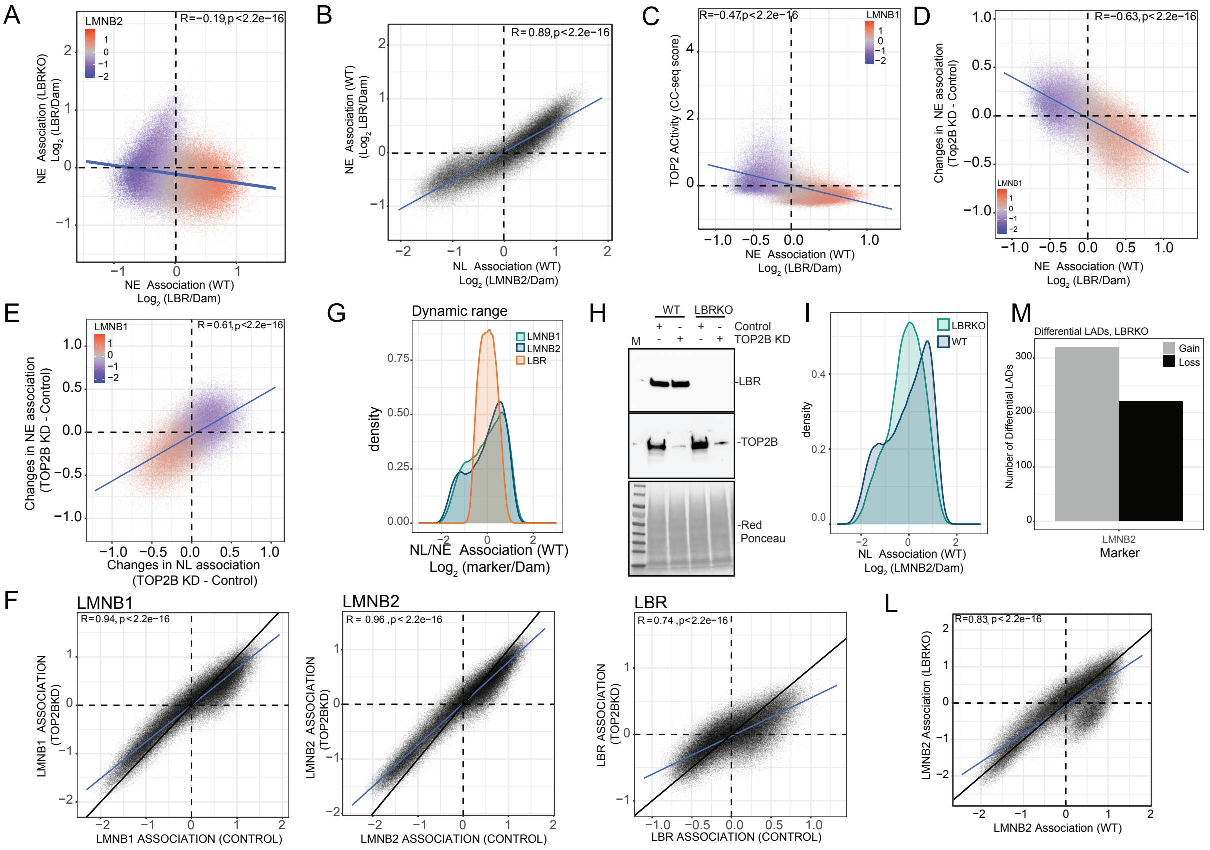

Supplementary Figure 8. Mapping of genome-LBR interactions in control, TOP2B-depleted and LBRKO cells. A) Correlation scatter plot of 20 kb genomic bins for LBR signal between Control and LBR knockout RPE1 cells. B) Correlation scatter plot of 20 kb genomic bins for pA-DamID score for LMNB2 and LBR mapping in WT RPE1 cells. C) Correlation scatter plot of 20 kb genomic bins between LBR association and TOP2 activity in RPE1 cells. D) Correlation scatters plot of 20 kb genomic bins for LBR association score (x-axis) and differential LBR score (y-axis) for TOP2B depletion (TOP2BKD - control). E) Correlation scatter plot of 20 kb genomic bins for differential LMNB2 and LBR scores (TOP2B knockdown - control). F) Correlation scatter plot of 20 kb genomic bins for LMNB1, LMNB2 and LBR scores (Control vs TOP2B knockdown). G) Density plot showing dynamic ranges for LMNB1, LMNB2 and LBR mapping in RPE1 cells. H) Western blot showing the level of LBR and TOP2B in Control, TOP2B KD, LBR knockout, and co-depletion TOP2B and LBR. Red ponceau is used as loading control. I) Density plot showing LMNB2 signal distribution in WT and LBR knockout cells. L) Correlation scatter plot of 20 kb genomic bins for pA-DamID score for LMNB2 between Control and LBR knockout RPE1 cells. For all correlation scatter plots: the blue line represents a linear model; the black line represents the diagonal; Pearson correlation and *P* value are shown in the plot. For A-I and L, results are from at least three biological replicates. For G, results are from two biological replicates and 4 technical replicates generated using two negative control siRNAs and two TOP2B-specific siRNAs. M) Results of Voom-Limma analysis used to call LADs that significantly gain or lose interaction with LMNB2 following LBR depletion. Results are from four biological replicates.

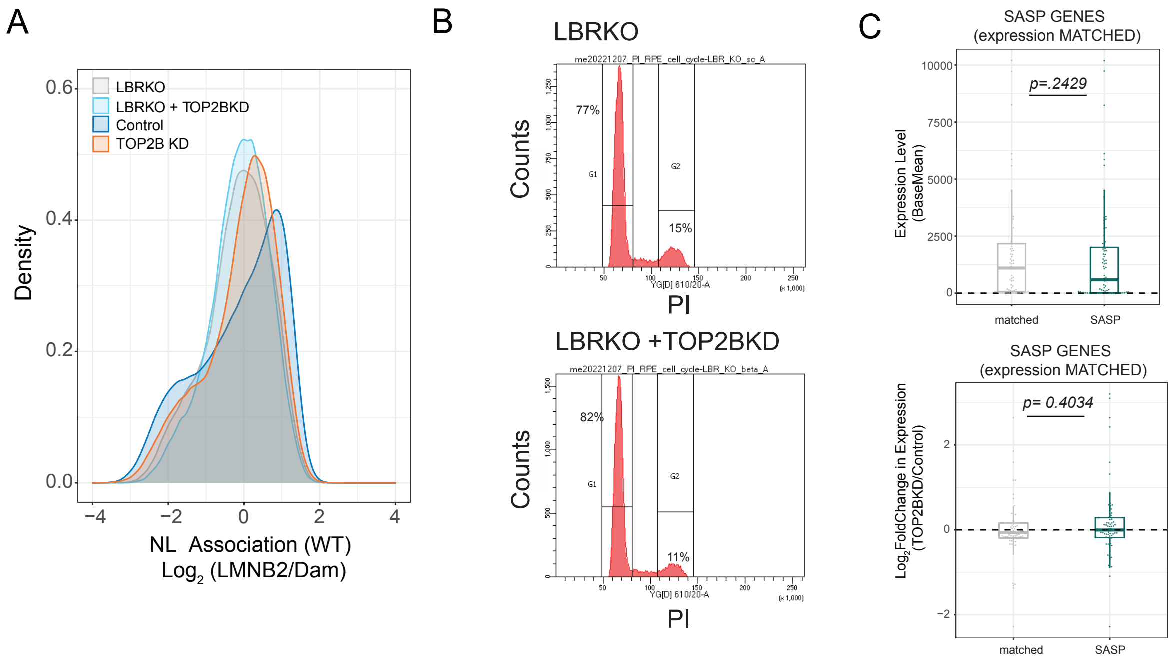

Supplementary Figure 9. Co-depletion of TOP2B and LBR and effect of TOP2B depletion on SASP genes transcription. A) Density plot showing LMNB2 signal distribution in WT, TOP2B depleted, LBR knockout and co-depleted (TOP2B knockdown + LBR knockout) RPE1 cells. B) Cell cycle analysis in LBR knockout cells with and without TOP2B, by PI staining. C) *Bottom panel*: changes in expression levels following TOP2B depletion for 80 SASP genes (https://reactome.org/PathwayBrowser/#/R-HSA-2559582). As a control we used a random set of genes that matched SASP genes for expression level (*top panel*). Matching for gene expression was performed as in (13)

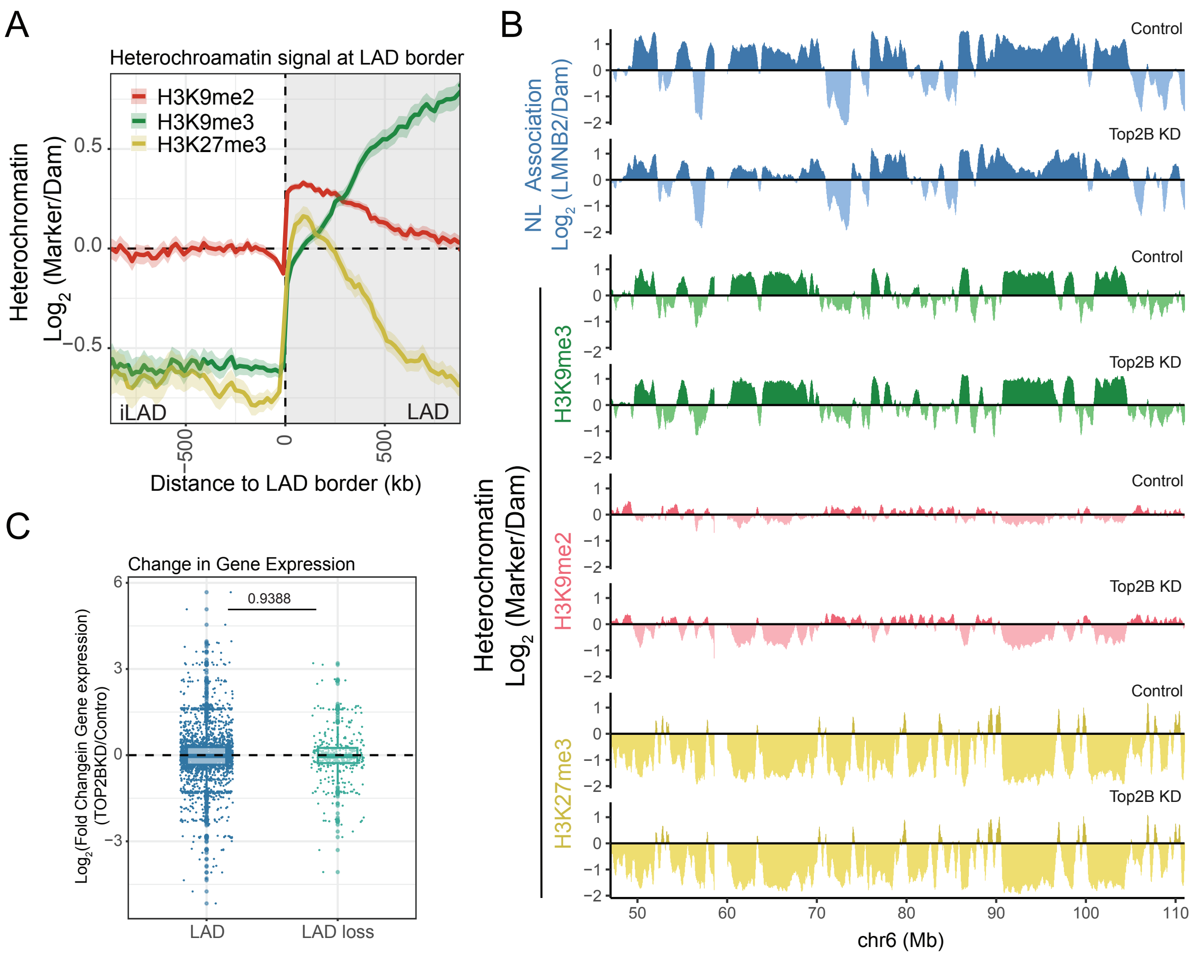

Supplementary Figure 10. A) Average H3K9me3, H3K9me2 and H3K27me3 pA-DamID scores around LAD borders for WT RPE1 cells. The solid line and the shaded area represent the mean signal and 95% confidence interval of the mean, respectively. B) Example genomic track for pA-DamID signal for LMNB2 (blue), H3K9me3 (green), H3K9me2 (red), and H3K27me3 (yellow) for Control and TOP2B depleted cells. 20KB bins were used. The antibody signal is normalized over a Dam-only control. Results are the average of two independent biological replicates. C) Change in gene expression following TOP2B depletion for genes in all LADs (blue) and in LADs that preferentially lose contact with LBR (light blue). *P* value is according to two-sided Wilcoxon's test. Results are the average of two independent biological replicates.

REFERENCES

1. Kundaje, A., Kyriazopoulou-Panagiotopoulou, S., Libbrecht, M., Smith, C.L., Raha, D., Winters, E.E., Johnson, S.M., Snyder, M., Batzoglou, S. and Sidow, A. (2012) Ubiquitous heterogeneity and asymmetry of the chromatin environment at regulatory elements. *Genome Res*, **22**, 1735-1747.

2. Schwartz, U., Nemeth, A., Diermeier, S., Exler, J.H., Hansch, S., Maldonado, R., Heizinger, L., Merkl, R. and Langst, G. (2019) Characterizing the nuclease accessibility of DNA in human cells to map higher order structures of chromatin. *Nucleic Acids Res*, **47**, 1239-1254.

3. Zheng, D., Trynda, J., Sun, Z. and Li, Z. (2019) NUCLIZE for quantifying epigenome: generating histone modification data at single-nucleosome resolution using genuine nucleosome positions. *BMC Genomics*, **20**, 541.

4. Descostes, N., Heidemann, M., Spinelli, L., Schuller, R., Maqbool, M.A., Fenouil, R., Koch, F., Innocenti, C., Gut, M., Gut, I. *et al.* (2014) Tyrosine phosphorylation of RNA polymerase II CTD is associated with antisense promoter transcription and active enhancers in mammalian cells. *Elife*, **3**, e02105.

5. Lavender, C.A., Shapiro, A.J., Burkholder, A.B., Bennett, B.D., Adelman, K. and Fargo, D.C. (2017) ORIO (Online Resource for Integrative Omics): a web-based platform for rapid integration of next generation sequencing data. *Nucleic Acids Res*, **45**, 5678-5690.

6. Porubský, D., Sanders, A.D., van Wietmarschen, N., Falconer, E., Hills, M., Spierings, D.C., Bevova, M.R., Guryev, V. and Lansdorp, P.M. (2016) Direct chromosome-length haplotyping by single-cell sequencing. *Genome Res*, **26**, 1565-1574.

7. Sanders, A.D., Meiers, S., Ghareghani, M., Porubsky, D., Jeong, H., van Vliet, M., Rausch, T., Richter-Pechanska, P., Kunz, J.B., Jenni, S. *et al.* (2020) Single-cell analysis of structural variations and complex rearrangements with tri-channel processing. *Nat Biotechnol*, **38**, 343-354.

8. Lenain, C., de Graaf, C.A., Pagie, L., Visser, N.L., de Haas, M., de Vries, S.S., Peric-Hupkes, D., van Steensel, B. and Peeper, D.S. (2017) Massive reshaping of genome-nuclear lamina interactions during oncogene-induced senescence. *Genome Res*, **27**, 1634-1644.

9. Meuleman, W., Peric-Hupkes, D., Kind, J., Beaudry, J.B., Pagie, L., Kellis, M., Reinders, M., Wessels, L. and van Steensel, B. (2013) Constitutive nuclear lamina-genome interactions are highly conserved and associated with A/T-rich sequence. *Genome Res*, **23**, 270-280.

10. Voong, L.N., Xi, L., Sebeson, A.C., Xiong, B., Wang, J.P. and Wang, X. (2016) Insights into Nucleosome Organization in Mouse Embryonic Stem Cells through Chemical Mapping. *Cell*, **167**, 1555-1570 e1515.

11. Yao, Q., Zhu, L., Shi, Z., Banerjee, S. and Chen, C. (2025) Topoisomerase-modulated genome-wide DNA supercoiling domains colocalize with nuclear compartments and regulate human gene expression. *Nat Struct Mol Biol*, **32**, 48-61.

12. Gittens, W.H., Johnson, D.J., Allison, R.M., Cooper, T.J., Thomas, H. and Neale, M.J. (2019) A nucleotide resolution map of Top2-linked DNA breaks in the yeast and human genome. *Nat Commun*, **10**, 4846.

13. Manzo, S.G., Mazouzi, A., Leemans, C., van Schaik, T., Neyazi, N., van Ruiten, M.S., Rowland, B.D., Brummelkamp, T.R. and van Steensel, B. (2024) Chromatin protein complexes involved in gene repression in lamina-associated domains. *EMBO J*, **43**, 5260-5287.
